# Global, regional, and national burden of alopecia areata in adolescents and young adults aged 10–24 years from 1990 to 2021: a trend analysis

**DOI:** 10.3389/fpubh.2026.1731022

**Published:** 2026-02-17

**Authors:** Su Liang, Yicheng Zhang, Xuesong Jia, Xue Xia, Xue Wang

**Affiliations:** 1 Department of Dermatology, The First Affiliated Hospital of Shihezi University, Shihezi, China; 2 Shihezi University School of Medicine, Shihezi, China; 3 Quality Control Department, The First Affiliated Hospital of Shihezi University, Shihezi, China

**Keywords:** alopecia areata, average annual percentage of change, disability-adjusted life years, global burden of disease, socioeconomic indices

## Abstract

**Objective:**

Alopecia areata (AA) is a common autoimmune hair loss disorder, posing a significant threat to the mental health and quality of life of patients, especially adolescents and young adults at critical stages of psychological and social development. This study conducted a comprehensive comparative analysis of the burden of AA in the global population aged 10–24 years, providing scientific basis for the prevention and treatment of AA.

**Methods:**

Using data from GBD 2021, AA prevalence, incidence, and disability-adjusted life years (DALYs) for individuals aged 10–24 years in 204 countries and territories from 1990 to 2021 were analyzed by age, sex, and region. Temporal trends were evaluated by average annual percentage of change (AAPC) calculated by the Joinpoint regression model.

**Results:**

From 1990 to 2021, the number of prevalence cases, incidence cases, and DALYs of AA among individuals aged 10–24 worldwide increased, however, the corresponding age-standardized rates all decreased, with AAPCs of −0.1374 (95% CI: −0.1396 to −0.1354), −0.1404 (95% CI: −0.1425 to −0.1387), and −0.1351 (95% CI: −0.1382 to −0.1317), respectively. The burden of AA in females was higher than that in males, and both decreased, with a more significant decrease in males; the 20–24 group had the largest rate decline but remained most burdened; the burden in high socioeconomic indices (SDI) regions was the highest, but the decrease was the most significant; the burden in North America was the highest, while the burden in South Asia, Western Europe, and North Africa and the Middle East regions showed an upward trend; the burden in the United States was the highest, but the decline was the greatest, and the increase was the largest in the United Arab Emirates.

**Conclusion:**

The absolute burden of AA among adolescents and young adults worldwide was still increasing, but the age-standardized rates had slightly decreased. There were significant age, gender, regional, and socioeconomic inequalities in the burden distribution. These differences should be fully considered, and comprehensive prevention, treatment, and psychological support measures should be adopted to continuously monitor and formulate precise strategies for AA in adolescents and young adults, reducing its burden and profound psychological impact.

## 1 Introduction

Alopecia areata (AA) is a chronic, specific autoimmune disease that affects hair follicles and leads to non scar hair loss, typically manifested clinically as patchy hair loss on the scalp. Epidemiological studies have shown that AA affects about 2% of the global population, with a lifetime risk of 1.7–2.1% (1), with higher prevalence in children and young people (2), and more common in females (3). The etiology of AA involves multiple factors such as genetics, immunity, and environment. This disease not only causes physical changes, but also has an impact on the patient’s mental health and overall quality of life. Studies have shown that patients with AA often experience significant psychological problems such as anxiety, depression, and decreased self-esteem, which may further lead to reduced participation in daily activities (4, 5).

In addition, patients with AA also have an increased risk of developing other diseases (5). For example, autoimmune diseases (such as atopic dermatitis, lupus erythematosus, psoriasis) and other potential comorbidities such as nutritional deficiency (such as vitamin D and iron), systemic diseases (such as diabetes) and cardiovascular diseases (such as atherosclerosis) further increase the burden of AA (5). The age group of 10–24 is considered a high-risk period for AA, although many studies have recognized that this age group is particularly affected, there is a lack of high-quality evidence to compare the burden of AA in different countries and territories, which is unfavorable for the formulation of effective measures.

In this study, we analyzed the prevalence, incidence, and disability-adjusted life years (DALYs) trend of AA among adolescents and young people at the global, regional, and national levels based on the data from GBD 2021. In addition, we conducted a stratified analysis of these trends by gender, region, and sociodemographic index (SDI). The findings contributed to a deeper understanding of the potential causes and related comorbidities of AA and were also helpful in designing prevention and intervention strategies for adolescents and young adults.

## 2 Methods

### 2.1 Study population and data collection

Data was sourced from the GBD 2021 database, which comprehensively collects data on 371 diseases from 204 countries and territories (1). All data are freely available through the Global Health Data Exchange platform.^1^ The original data sources and fitting methods for the GBD Study 2021 have been thoroughly detailed in a previous study (6). The GBD 2021 categorizes causes into four levels, ranging from level 1 infectious, maternal, neonatal, and nutritional diseases to level 4 latent tuberculosis infection. AA is listed as a Level 3 cause of the GBD 2021. In GBD 2021, AA is identified using International Classification of Diseases (ICD)-10 codes L63–L63.9 and ICD-9 codes 704.0–704.9 (6).

The United Nations has come to formally define adolescence as the period between 10 and 19 years of age (7). In order to investigate in more detail, we divided adolescents into younger adolescents (aged 10–14 years) and older adolescents (aged 15–19 years), in addition to young adults aged 20–24 years (8). We collected data for AA from both sexes across three age groups: 10–14, 15–19, and 20–24 years old. The numbers of prevalent cases, incident cases, and prevalence and incidence rates, in addition to the numbers and rates for DALYs, were directly extracted from the GBD 2021. DALYs were obtained by calculating the sum of the years of life lost and years lived with disability (6). The Socio-demographic Index (SDI) is a composite indicator of background social and economic conditions that influence health outcomes in each location. It is the geometric mean of 0 to 1 indices of total fertility rate for those younger than 25 years old, mean education for those 15 years old and above, and lag-distributed income per capita. The GBD 2021 categorizes countries and territories into five SDI quintiles ranging from low to high development levels (6).

### 2.2 Statistical analysis

To estimate the age-standardized prevalence rate (ASPR), incidence rate (ASIR), and DALY rate (ASDR) of AA among adolescents and young adults, age standardization was performed using the direct method, which assumes the rates distribution is a weighted sum of independent Poisson random variables (9). By using these standardized metrics to quantify the differences in the burden of AA across various time periods, sex, and locations, the impact of variations in population age structures can be controlled. All rates were reported per 100,000 population. The 95% uncertainty intervals (UIs) were defined by the 25th and 75th values of the ordered 1,000 estimates based on the GBD algorithm.

The age-standardized rate calculation formula is as follows:

$$\mathrm{Age}-\text{standardized rate}=\frac{\sum_{i=1}^{A}a_{i}w_{i}}{\sum_{i=1}^{A}w_{i}}$$

Where, $a_{i}$ is the age-specific rate for the *i*th age group, and $w_{i}$ is the weight in the corresponding age subgroup of the chosen reference standard population (where *i* denotes the *i*th age class), and *A* represents the total number of age groups.

The Joinpoint regression analysis is an effective method for analyzing trends over time to identify the time points at which the trend significantly changes. This model adeptly identifies and quantitatively characterizes significant change points within the time-series data concerning age-standardized rates of AA across global, regional, and national levels. The model facilitated the computation of the annual percent change (APC) and its accompanying 95% confidence interval (CI) to delineate age-standardized rates trends across delineated time frames (10). Moreover, for a holistic appraisal of the observed trends, the average annual percent change (AAPC) was also calculated, encapsulating aggregated trend data spanning the study period of 1990–2021. The AAPC calculation formula is as follows:

$$\text{AAPC}=\{\exp(\frac{\sum_{i=1}^{B}w_{i}b_{i}}{\sum_{i=1}^{B}w_{i}})-1\}\times 100$$

Where, $b_{i}$ is the slope coefficient for the *i* th segment, with *i* indexing the segments within the desired range of years, and $w_{i}$ represents the length of each segment within that range, and *B* demotes the total number of segments.

From a statistical standpoint, an APC or AAPC estimate, alongside its 95% CI lower bound exceeding zero, denotes an upward trajectory in the specified interval. In contrast, an APC or AAPC estimate coupled with a 95% CI upper bound falling below zero signals a downward trend. When the 95% CI for the APC or AAPC encompasses zero, it implies that the trend has remained stable. All statistical analyses were conducted using the Joinpoint Regression Program (version 5.0.2) and R (version 4.3.2). Two-tailed *p* < 0.05 was considered statistically significant.

## 3 Results

### 3.1 Global trends in alopecia areata

The global number of prevalence cases of AA among adolescents and young adults worldwide had increased by 16.46% compared to 1990, from 2712439.2 (95% UI: 2512374.3 to 2924044.1) in 1990 to 3,158,846 (95% UI: 2926498.6 to 3395234.3) in 2021 (Table 1). In addition, from 1990 to 2021, the ASPR of AA decreased from 173.8 (95% UI: 156.4 to 192.1) per 100,000 people to 166.5 (95% UI: 149.9 to 183.7) per 100,000 people, with an AAPC of −0.1374 (95% CI: −0.1396 to −0.1354) (Table 1; Supplementary Figure S1). At the same time, the global number of incidence cases of AA in 2021 reached 5881661.5 (95% UI: 5453313.4 to 6356782.1), higher than the 5055219.9 (95% CI: 4681475.9 to 5465260.6) in 1990 (Supplementary Table S1). The ASIR showed a downward trend, with an AAPC of −0.1404 (95% CI: −0.1425 to −0.1387), decreasing from 323.9 (95% UI: 288.8 to 360) per 100,000 people in 1990 to 310 (95% UI: 276.8 to 344.5) per 100,000 people in 2021 (Supplementary Table S1; Supplementary Figure S2). The DALYs caused by AA increased from 90,353 (95% UI: 57820.5 to 129223.3) in 1990 to 105334.5 (95% UI: 67557.2 to 150574.7) in 2021, with an increase of 16.58% (Supplementary Table S2). ASDR followed a declining trend, decreasing from 5.8 (95% UI: 3.7 to 8.4) per 100,000 people in 1990 to 5.6 (95% UI: 3.6 to 8.1) per 100,000 people in 2021, with an AAPC of −0.1351 (95% CI: −0.1382 to −0.1317) (Supplementary Table S2; Supplementary Figure S3).

**Table 1:** Age-standardized prevalence and AAPC of alopecia areata in adolescents and young adults aged 10–24 years at global and regional level, 1990–2021.

Groups	Prevalence (95% UI)				
	Cases in 1990	Age-standardized rate in 1990 (per 100,000)	Cases in 2021	Age-standardized rate in 2021 (per 100,000)	AAPC (95% CI)
Global	2712439.2 (2512374.3 to 2924044.1)	173.8 (156.4 to 192.1)	3,158,846 (2926498.6 to 3395234.3)	166.5 (149.9 to 183.7)	−0.1374 (−0.1396 to −0.1354)
Sex					
Female	1690348.5 (1566321.8 to 1823232.5)	219.6 (197.4 to 243)	1967153.9 (1820558.2 to 2117850.5)	212.2 (190.7 to 234.3)	−0.1108 (−0.1126 to −0.1089)
Male	1022090.7 (944800.7 to 1101680.8)	129.2 (116.2 to 143.2)	1191692.2 (1103501.4 to 1280836.8)	122.8 (110.5 to 136)	−0.1615 (−0.1641 to −0.1588)
SDI level					
High SDI	434,996 (402509.7 to 466983.5)	213.1 (192 to 234.7)	396176.9 (368111.4 to 424893.8)	206.1 (185.7 to 226.7)	−0.1051 (−0.1158 to −0.0938)
High-middle SDI	520118.7 (480671.5 to 563236.4)	177 (158.5 to 197.4)	400714.5 (371638.4 to 432860.2)	174.6 (156.6 to 194.2)	−0.0444 (−0.0455 to −0.0429)
Middle SDI	974600.2 (900,623 to 1055017.3)	174.5 (156.5 to 193.9)	946465.6 (875590.1 to 1021085.1)	169.7 (152.8 to 187.7)	−0.0893 (−0.0904 to −0.0885)
Low-middle SDI	548932.2 (509100.3 to 590,384)	155.2 (139 to 171.8)	855930.4 (793,180 to 919,748)	153.9 (137.8 to 170.5)	−0.0268 (−0.0273 to −0.0263)
Low SDI	231447.3 (214,943 to 248,247)	154.4 (138.6 to 170.5)	557,098 (517278.1 to 597990.4)	154.6 (138.8 to 171.1)	0.0050 (0.0047 to 0.0053)
GBD region					
Central Asia	32173.6 (29633.1 to 34764.7)	163.3 (145.3 to 182.2)	35738.1 (32940.4 to 38627.4)	162.3 (144.3 to 180.9)	−0.0209 (−0.0214 to −0.0205)
South Asia	475837.5 (439332.8 to 512730.7)	144.7 (129.2 to 160.8)	773981.7 (714909.8 to 834171.2)	144.9 (129.3 to 161.1)	0.0039 (0.0036 to 0.0043)
Southeast Asia	300843.7 (278,332 to 324990.8)	204.8 (183.1 to 227.7)	354091.9 (326842.7 to 382702.8)	203.5 (182.1 to 226.6)	−0.0198 (−0.0200 to −0.0195)
East Asia	711614.5 (653,783 to 777279.5)	180.5 (160.3 to 202.6)	432404.7 (399003.6 to 469230.7)	178.1 (158.2 to 199.8)	−0.0434 (−0.0444 to −0.0424)
High-income Asia Pacific	88388.6 (81712.1 to 95184.8)	203.7 (182 to 226.4)	55,758 (51560.3 to 60089.3)	203.6 (182 to 226.3)	−0.0007 (−0.0009 to −0.0006)
Western Europe	171257.1 (157537.3 to 184442.8)	197.1 (177 to 218.8)	146055.3 (134652.8 to 157059.9)	197.2 (176.9 to 218.6)	0.0012 (0.0011 to 0.0012)
Central Europe	47398.5 (43867.7 to 50871.3)	162.9 (146.1 to 181.1)	29906.6 (27638.2 to 32,141)	162.5 (145.6 to 180.4)	−0.0087 (−0.0089 to −0.0084)
Eastern Europe	77846.1 (71785.4 to 84115.7)	163.8 (145.7 to 182.6)	53137.7 (48955.9 to 57428.6)	163.3 (145.3 to 182.2)	−0.0082 (−0.0084 to −0.0080)
Central Sub-Saharan Africa	26,386 (24497.5 to 28437.4)	156.9 (140.2 to 175)	68175.6 (63306.3 to 73470.7)	156.9 (140.1 to 174.9)	−0.0014 (−0.0016 to −0.0011)
Eastern Sub-Saharan Africa	94,709 (87774.8 to 101676.1)	159.3 (142.5 to 176.8)	225213.3 (208988.8 to 242591.3)	158.6 (141.6 to 176.1)	−0.0148 (−0.0159 to −0.0138)
Southern Sub-Saharan Africa	26754.4 (24769.5 to 28921.9)	159 (142.1 to 176.7)	34254.4 (31704.7 to 37015.9)	157.4 (140.8 to 174.7)	−0.0329 (−0.0332 to −0.0327)
Western Sub-Saharan Africa	92,477 (85586.1 to 99607.9)	159.7 (143.1 to 177.2)	248102.1 (229635.8 to 267173.5)	159 (142.5 to 176.5)	−0.0128 (−0.0135 to −0.0121)
North Africa and Middle East	155785.4 (144023.1 to 168532.6)	146.6 (130.8 to 163.3)	237212.8 (219648.5 to 256,587)	146.6 (130.7 to 163.3)	0.0004 (−0.0001 to 0.0008)
Andean Latin America	19,856 (18288.2 to 21439.5)	164.2 (146 to 183.2)	28607.9 (26347.3 to 30933.2)	162.1 (144.2 to 180.7)	−0.0420 (−0.0427 to −0.0414)
Tropical Latin America	78090.5 (71851.8 to 84336.5)	165 (146.9 to 184.6)	86058.1 (79135.1 to 93180.4)	164 (146 to 183.3)	−0.0200 (−0.0202 to −0.0198)
High-income North America	166001.9 (154680.6 to 177376.9)	260 (235.8 to 285.1)	170785.7 (159752.1 to 182563.8)	233.3 (211.2 to 255.9)	−0.3428 (−0.3735 to −0.3075)
Caribbean	17786.6 (16373.3 to 19,243)	164.1 (145.9 to 183.1)	18,888 (17390.7 to 20428.8)	163.1 (145.1 to 182)	−0.0194 (−0.0198 to −0.0190)
Central Latin America	88201.4 (81738.9 to 94856.5)	165.2 (147.9 to 183.4)	108574.4 (100529.5 to 116892.5)	164 (146.9 to 182.1)	−0.0225 (−0.0227 to −0.0222)
Southern Latin America	26991.4 (24861.1 to 29092.3)	205 (182.5 to 228.1)	32320.7 (29736.8 to 34,887)	204.8 (182.2 to 227.8)	−0.0045 (−0.0046 to −0.0043)
Oceania	3879.9 (3571.7 to 4206.4)	188.7 (168.7 to 211)	7548.5 (6946.2 to 8199.3)	188 (168.2 to 210.2)	−0.0118 (−0.0124 to −0.0114)
Australasia	10160.2 (9387.9 to 10966.3)	204.9 (183.2 to 227.9)	12030.5 (11128.9 to 12982.8)	204.8 (183 to 227.7)	−0.0027 (−0.0028 to −0.0026)

AAPC, Average annual percentage change.

### 3.2 Global trends of alopecia areata stratified by gender

**Figure 1:**
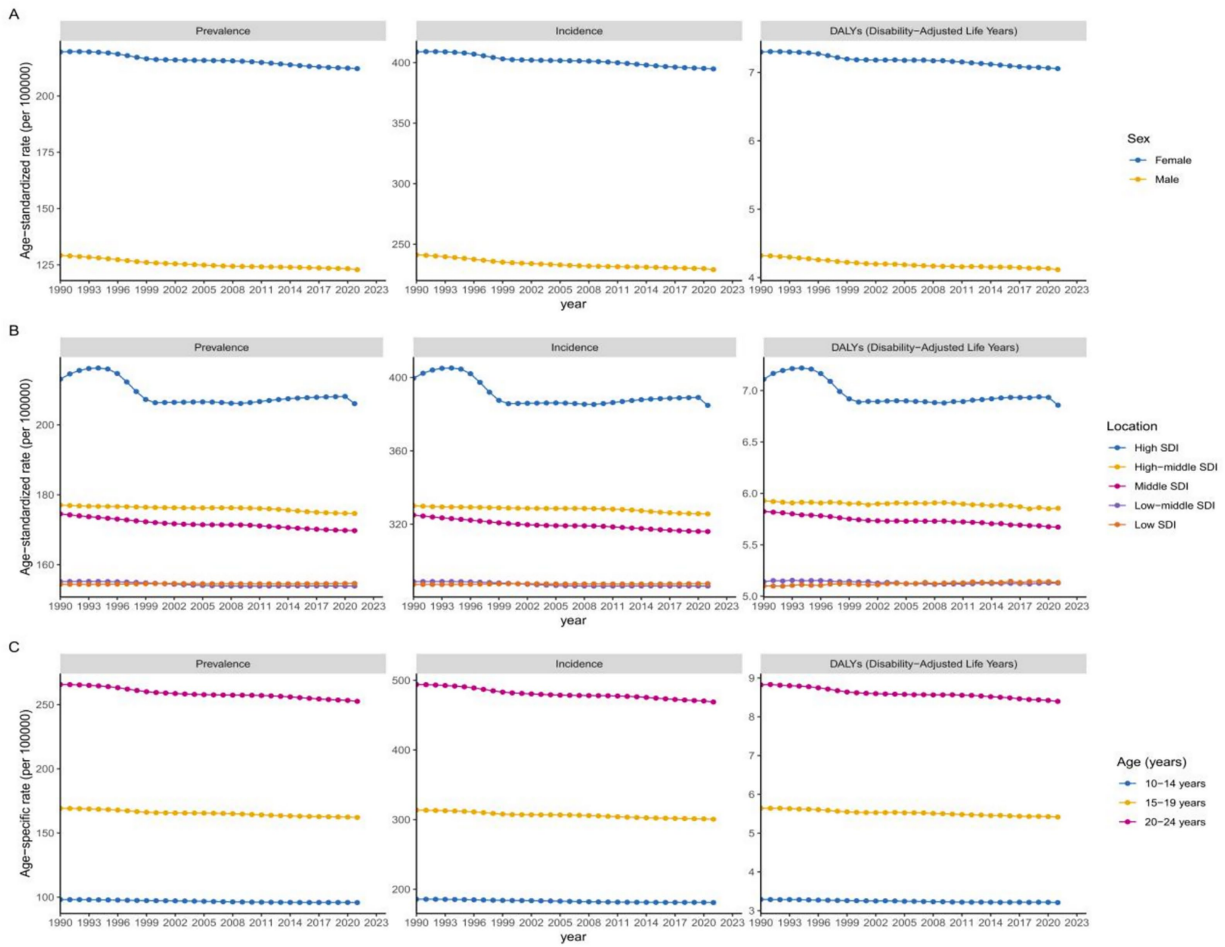
Global trends by gender **(A)**, SDI quintiles **(B)** for ASPR, ASIR, and ASDR of alopecia areata in adolescents and young adults aged 10–24 years from 1990 to 2021. Global trends by age groups **(C)** for age-specific prevalence rate, incidence rate, and DALY rate of alopecia areata in adolescents and young adults from 1990 to 2021. SDI, socio-demographic index; ASPR, age-standardized prevalence rate; ASIR, age-standardized incidence rate; ASDR, age-standardized DALY rate; DALYs, disability adjusted life-years.

The burden of AA among young males and females worldwide had decreased from 1990 to 2021 (Figure 1A). In 2021, the ASPR of young females was about 72.8% higher than that of young males (212.2 per 100,000 vs. 122.8 per 100,000) (Table 1). In the past 30 years, the gender gap in ASPR had slightly increased, with a more pronounced decline in male patients (AAPC: -0.1615, 95% CI: −0.1641 to −0.1588) compared to females (AAPC: -0.1108, 95% CI: −0.1126 to −0.1089) (Table 1). Similarly, the ASIR of young females (AAPC: -0.1125, 95% CI: −0.1143 to −0.1105) and young males (AAPC: -0.1673, 95% CI: −0.1700 to −0.1643) showed a decreasing trend (Supplementary Table S1). The ASDR of male and female patients also showed a decreasing trend, with AAPC of −0.1085 (95% CI: −0.1122 to −0.1046) for young females and −0.1568 (95% CI: −0.1644 to −0.1494) for young males (Supplementary Table S2).

### 3.3 Global trends of alopecia areata stratified by age

The age specific prevalence rate, incidence rate and DALYs rate of AA in 2021 were mainly concentrated in young adults aged 20–24 (Figure 1C). The decline in age specific prevalence rate among the global 20–24 age group was most significant from 1990 to 2021 (AAPC: -0.1628, 95% CI: −0.1660 to −0.1597). The prevalence rates of individuals aged 10–14 and 15–19 also decreased during this period, with AAPCs of −0.1384 (95% CI: −0.1403 to −0.1364) and −0.0764 (95% CI: −0.0778 to −0.0748), respectively (Supplementary Table S3). In addition, from 1990 to 2021, the age specific incidence rate of the 20–24 age group declined the most, with the AAPC of −0.1691 (95% CI: −0.1714 to −0.1667). The age specific DALYs rate of AA among the 10–24 age group also declined (Supplementary Table S3).

### 3.4 Global trends of alopecia areata stratified by SDI quintiles

From 1990 to 2021, except for low SDI regions, the burden of AA in other regions had shown a decreasing trend over time (Figure 1B). From 1990 to 2021, the low SDI regions gradually increased and surpassed the low-middle SDI regions over time, while the high SDI regions had the highest ASPR, ASIR, and ASDR for AA (Figure 1B). Although regions with high SDI had the highest burden of AA, during this period, the ASPR, ASIR, and ASDR in these regions decreased the most compared to other SDI regions, with AAPCs of −0.1051 (95% CI: −0.1158 to −0.0938), −0.1180 (95% CI: −0.1292 to −0.1059), and −0.1144 (95% CI: −0.1268 to −0.1017), respectively (Table 1; Supplementary Tables S1, S2). In addition, from 1990 to 2021, ASPR, ASIR, and ASDR in high-middle SDI regions ranked second, while ASPR, ASIR, and ASDR in middle SDI regions showed the second largest decline. Only ASPR, ASIR, and ASDR in low SDI regions showed an upward trend, with AAPCs of 0.0050 (95% CI: 0.0047 to 0.0053), 0.0049 (95% CI: 0.0047 to 0.0052), and 0.0266 (95% CI: 0.0224 to 0.0307), respectively (Table 1; Supplementary Tables S1, S2).

### 3.5 Regional trends of alopecia areata

**Figure 2:**
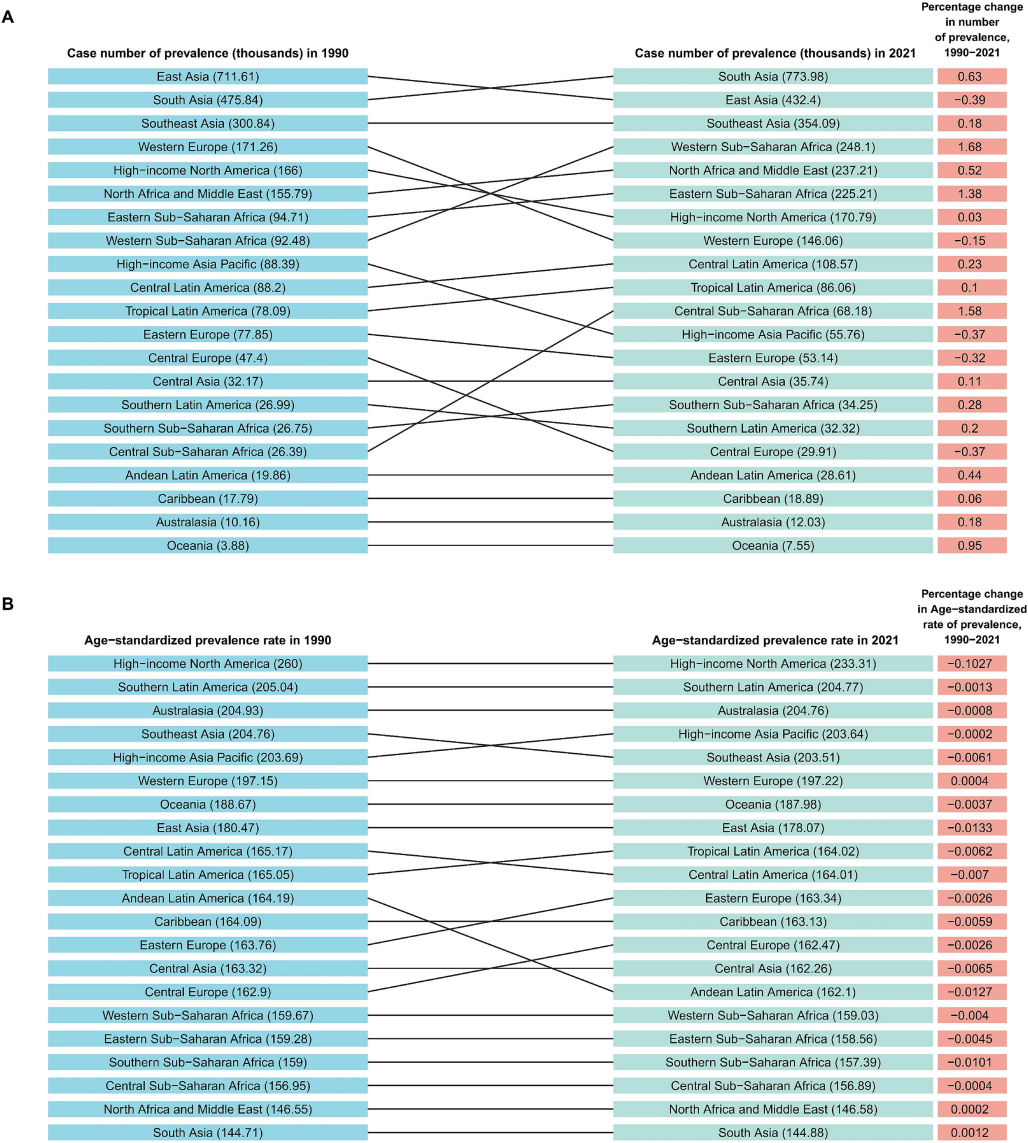
The number of prevalent cases **(A)** and ASPR **(B)** for alopecia areata among adolescents and young adults across 21 GBD regions from 1990 to 2021. GBD, Global Burden of Disease; ASPR, Age-standardized prevalence rate.

Based on the 21 regions partitioned by the GBD 2021 database, we analyzed the differential characteristics of ASPR, ASIR, and ASDR in adolescents and young adults AA in each region (Figure 2; Supplementary Figures S4, S5). South Asia reported very high numbers in 2021, with 1,438,950 prevalence cases, 773981.7 incidence cases, and 25782.5 DALYs. Although South Asia had the highest prevalence cases and incidence cases, in terms of standardized rates, high-income North America had the highest ASPR (233.3 per 100,000 people), ASIR (435.2 per 100,000 people), and ASDR (7.7 per 100,000 people) in 2021. The ASPR (144.9 per 100,000 people), ASIR (269.4 per 100,000 people), and ASDR (4.8 per 100,000 people) in South Asia were the lowest. From 1990 to 2021, except for South Asia, Western Europe, North Africa, and the Middle East, ASPR and ASIR in other GBD regions showed a downward trend (AAPCs<0). The ASDR in South Asia, Western Europe, sub Saharan Africa, sub Saharan Africa, North Africa, and the Middle East showed an upward trend (AAPCs>0), while in other regions it showed a downward trend (AAPCs<0) (Table 1; Supplementary Tables S1, S2).

### 3.6 National trends in alopecia areata

**Figure 3:**
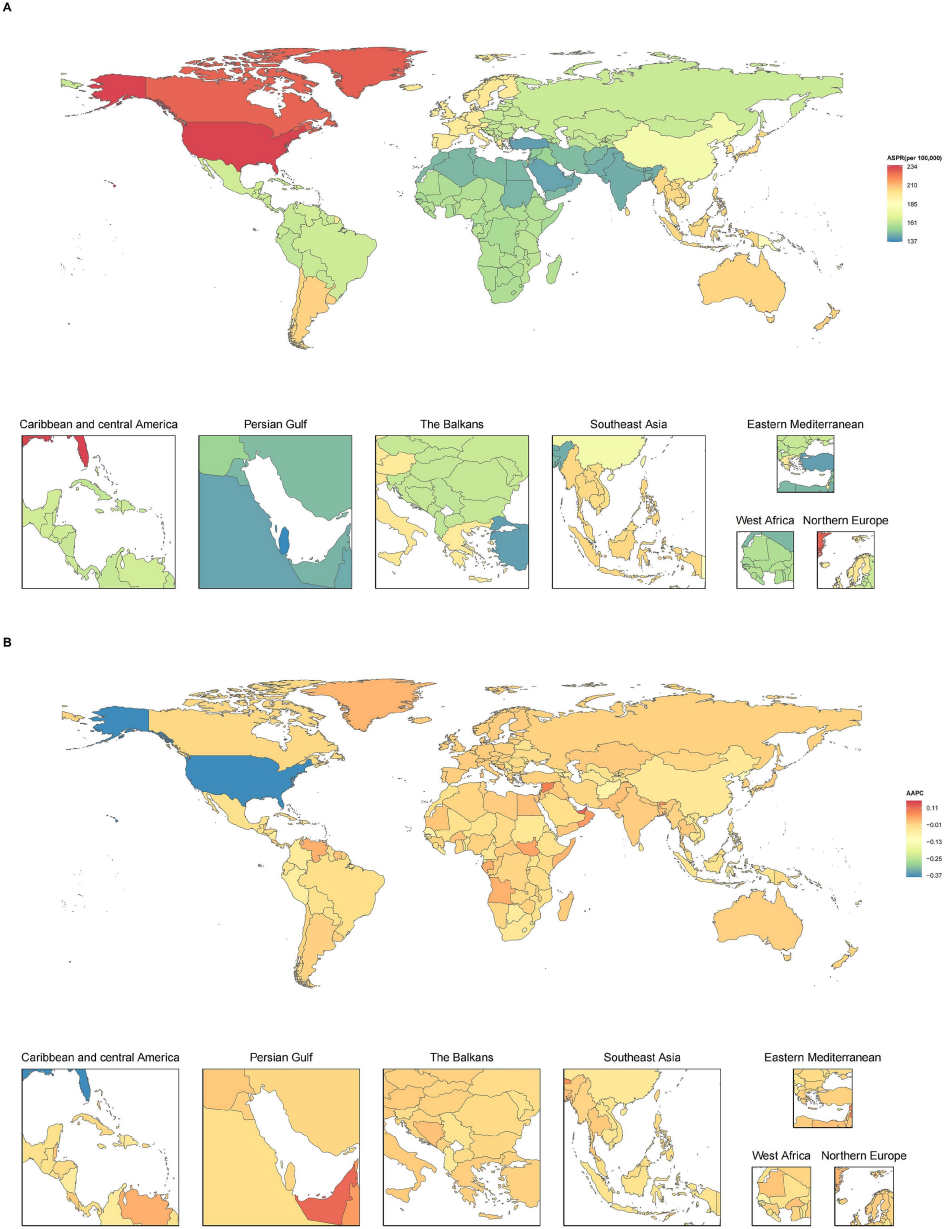
The global prevalence of alopecia areata among adolescents and young adults aged 10–24 years in 204 countries and territories. **(A)** ASPR in 2021. **(B)** AAPC in prevalence from 1990 to 2021. AAPC: Average annual percentage change; ASPR, age-standardized prevalence rate.

The top five countries in terms of ASPR (per 100,000 people) for AA in 2021 were the United States (233.90), Greenland (228.46), Canada (227.49), New Zealand (205.75), and Argentina (204.85) (Figure 3A; Supplementary Table S4). From 1990 to 2021, the United States had the largest decline in ASPR (AAPC: −0.3747), while the United Arab Emirates had the largest increase (AAPC: 0.1141) (Figure 3B). The top five countries in terms of ASIR (per 100,000 people) for AA were the United States (436.08), Greenland (427.80), Canada (426.13), New Zealand (384.40), and Argentina (383.48). The country with the lowest ASIR was Qatar (254.43) (Supplementary Figure S6; Supplementary Table S4). From 1990 to 2021, the United Arab Emirates had the largest increase in ASIR (AAPC: 0.1138), while the United States had the largest decrease (AAPC: −0.4081) (Supplementary Figure S6). The ASDR for AA in 2021 was the highest in the United States (7.75 per 100,000), and Qatar had the lowest (4.55 per 100,000) (Supplementary Figure S7; Supplementary Table S4). From 1990 to 2021, the United Arab Emirates had the largest increase in ASDR (AAPC: 0.1024), while the United States had the largest decrease (AAPC: −0.3930) (Supplementary Figure S7).

### 3.7 Correlation between alopecia areata and 21 comorbidities

**Figure 4:**
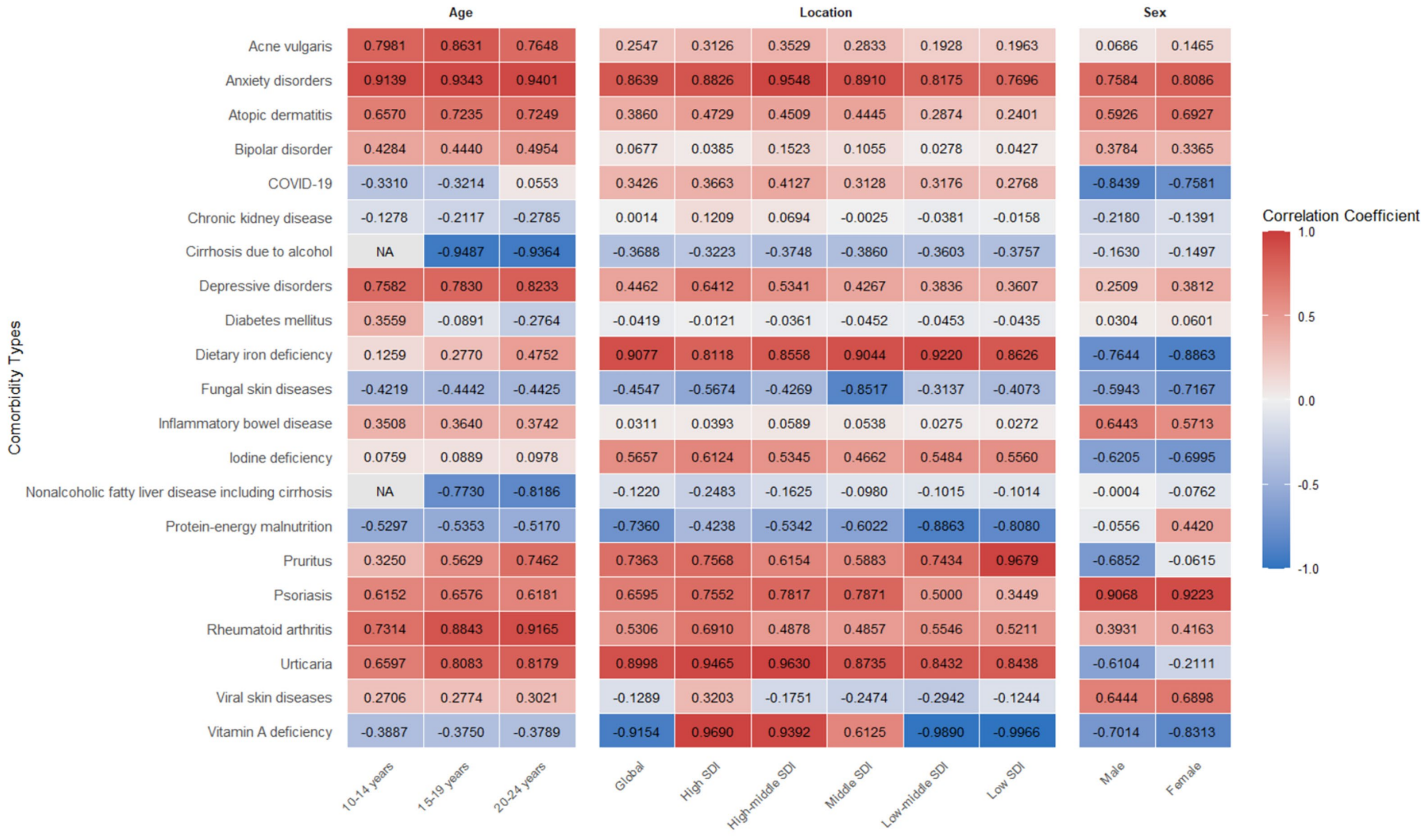
Correlation heatmap of potential comorbidities with alopecia areata prevalence across regions and demographic groups (age and sex). SDI, Socio-Demographic Index.

On a global scale, AA was significantly positively correlated with various diseases, such as anxiety disorders, atopic dermatitis, depressive disorders, dietary iron deficiency, rheumatoid arthritis, and urticaria, etc. Among the three age groups of 10–24 years, AA showed a stronger correlation with anxiety disorders, followed by depressive disorders, rheumatoid arthritis, and urticaria. From the perspective of gender, both men and women with AA had a stronger correlation with psoriasis, followed by anxiety disorders. In regions with a higher SDI, both anxiety disorders and depressive disorders showed a stronger correlation with AA, while in regions with a lower SDI, the correlation between psoriasis and AA was more obvious (Figure 4).

## 4 Discussion

Based on GBD 2021 data, this study focused on adolescents and young adults aged 10–24 years, and systematically analyzed the global, regional and national prevalence, incidence and DALYs of AA by age, gender and regions from 1990 to 2021. The results showed that although the absolute number of AA cases was still increasing, the global age-standardized prevalence rate, incidence rate, and DALYs rate of AA had declined. This indicated that although the global burden of AA was still increasing, the growth rate had slowed down.

This study found that the 20–24 age group had the highest burden of AA. This was consistent with another study, which showed that the peak incidence of AA was concentrated in youth, and 20–34 years was the age group with the highest incidence rate and DALYs rate (11). Although the relevant indicators of AA in the 10–19 age group were lower than that of the 20–24 age group, their burden was still significant, and the downward trend was weaker than that of the 20–24 age group. At the same time, they are in a critical period of personal psychological, social, and professional development, so the negative impact they brought is particularly profound and need to be given special attention. AA was closely related to psychological comorbidities such as anxiety, depression, and behavioral disorders, and this association was particularly prominent in children and adolescents (12–14). Adolescence and early adulthood are critical stages for self-identity and social relationship formation. Significant changes in appearance can lead to severe psychological distress, social phobia, social dysfunction, and even increase the risk of self harm. Although some mild psychological symptoms that may occur during childhood may alleviate with individual growth (15), AA itself and the stressful life events it brings may become important factors in inducing or exacerbating adolescent mental and psychological disorders (15, 16). In summary, the management of AA in the 10–24 age group should not only focus on clinical symptom control, but also its psychological and social dimensions are equally crucial.

The burden of AA in females continued to be higher than that in males. Although both burdens had decreased, the decrease was more pronounced in males, and the gender gap continued to widen. Therefore, the stress level caused by AA on females was much higher than that on males. According to another study, severe AA (hair loss exceeding 50% of the scalp area) was more common in females (17). This gender difference also existed in adolescents and young adults, and may be related to female specific immune regulatory mechanisms, hormone fluctuations (during puberty), and higher susceptibility to autoimmune diseases (18, 19). Changes in the endocrine environment to some extent affected pro-inflammatory and anti-inflammatory cytokines (20), as well as T cell activity (21), which in turn affected the autoimmune system, leading to a more severe burden on females.

From the perspective of global disease distribution, the burden of AA showed a high correlation with SDI. The SDI value of a region was negatively correlated with its age standardized prevalence rate, incidence rate, and DALYs rate. In high SDI regions such as the United States, the age standardization rate showed a significant downward trend due to high medical accessibility, multiple opportunities for early detection, strong disease awareness, and more advanced treatment methods. In addition, international attention to AA have been increasing year by year, and the establishment of the National AA Foundation and the emergence of different scoring scales and guidelines (such as the SALT score) for assessing the severity of the condition provide favorable conditions for the diagnosis and treatment of AA. However, the burden in these regions was still relatively high, and patients may have a stronger correlation with comorbidities such as depression due to facing greater social pressure and appearance anxiety (22). This study found that in the adolescent and young adult population of regions with a higher SDI, AA was more significantly associated with anxiety disorders and depressive disorders, which was consistent with the results of another study on AA in the entire population (23). A study assessing the bidirectional association between major depressive disorder and AA found that patients with major depression disorder had a 90% increased risk of developing AA, while individuals with AA had a 34% increased risk of developing major depression disorder (24). On the contrary, in regions with low SDI, although the rates related to AA were low, due to population growth, economic development and the gradual improvement of the medical diagnosis and reporting system, the age standardized rates showed a rapid upward trend. The nutritional deficiencies (such as vitamin D, zinc, folate, etc.) and high incidence of infection and inflammation faced by these regions might further exacerbate the risk and burden of AA (25, 26).

In summary, AA in adolescents and young adults aged 10–24 was a significant public health issue that could not be ignored, and it may be a comorbidity with other diseases such as atopic dermatitis or psoriasis (27). A survey conducted in the United States showed that patients with more severe AA not only had a higher incidence of atopic dermatitis, but also had a 78% increased risk of being diagnosed with atopic dermatitis. The results also showed that the prevalence and incidence rate of atopic dermatitis in adolescent patients with AA were significantly higher than those in adult patients regardless of the severity of the disease (28). Our study also found that among individuals aged 10–24, AA had a significant correlation with various diseases (anxiety disorders, atopic dermatitis, depression disorders, and urticaria). Therefore, the management of AA requires a multidimensional and individualized comprehensive strategy. Firstly, clinical diagnosis and treatment should go beyond simple hair development. Early identification and intervention of accompanying mental health issues are crucial, especially for female patients. Secondly, health policy makers should be aware of the differences in disease burden under different socio-economic backgrounds. In low SDI regions, disease screening, diagnostic system construction, and public health education should be strengthened, while in high SDI regions, support for patients’ social and psychological needs and investment in medical resources should be emphasized. Future studies should focus on exploring effective psychological interventions for this age group and optimizing treatment strategies to improve their long-term quality of life and prognosis.

Although this study provides important insights into the burden of AA in adolescents and young adults aged 10–24, there are still several limitations that needs to be addressed. This study is based on the analysis of the GBD database, whose data quality varies in different countries and territories. In low SDI regions with limited medical resources and incomplete disease surveillance systems may lead to insufficient diagnosis of AA, resulting in an underestimation of the true disease burden in these regions. This study do not differentiate between different clinical subtypes of AA (such as patchy, total, and generalized) and severe AA (hair loss area >50%) may bring more severe disease burden and psychological impact. The lack of stratified information limits the in-depth evaluation of the impact of disease severity. The impact of AA on the quality of life of adolescents and young adults is mainly reflected in the psychosocial level. The relevant indicators in this study (such as prevalence, incidence, and DALYs) cannot fully show the emotional distress, social function defects and economic burden caused by the disease, which may lead to an incomplete measurement of the overall impact of the disease. In addition, this study fails to include some individual level factors that may affect disease occurrence, such as genetic background, specific nutritional status, and lifestyle, and the absence of these confounding factors may affect the explanatory power of the results. Finally, adolescents and young adults at different developmental stages may face different disease manifestations and psychosocial challenges, and this limitation deserves further improvement in future study.

## 5 Conclusion

This study was based on GBD 2021, and for the first time comprehensively depicted the burden map of AA in adolescents and young adults worldwide from 1990 to 2021. Although the absolute number of AA cases continued to rise globally, the age-standardized rates all showed a downward trend, indicating that the growth rate of the burden was lower than the population growth, and the prevention and control work had achieved some results, but the increase of the absolute number was still an important public health problem. Especially for the age group of 10–24, AA is far more than just a superficial problem. Its psychological and social impacts, such as appearance anxiety, decreased self-esteem, and social disorders, are particularly severe, and it is a core component of the burden. For the management of AA in adolescents and young adults, comprehensive strategies should be adopted based on different genders and regions, including early diagnosis and individualized treatment, routine screening and intervention for comorbidities such as anxiety and depression, and the development of effective psychological and pharmacological intervention measures to improve their quality of life.

## Data Availability

Publicly available datasets were analyzed in this study. This data can be found at: https://ghdx.healthdata.org/gbd-2021/sources.

## Footnotes

1
https://ghdx.healthdata.org/gbd-2021/sources
