# Supplementary material for: Global, regional, and national burden of alopecia areata in adolescents and young adults aged 10–24 years from 1990 to 2021: a trend analysis
Source: Front Public Health. 2026 Feb 17;14:1731022. doi: 10.3389/fpubh.2026.1731022 (PMC12953509; doi:10.3389/fpubh.2026.1731022)
Supplement: Supplementary file 1 [file Table_1.DOCX]

| Table S1 Age-standardized incidence and AAPC of alopecia areata in adolescents and young adults aged 10-24 years at global and regional level, 1990-2021 | | | | | |
| --- | --- | --- | --- | --- | --- |
|  | Incidence (95% UI) |  |  |  |  |
|  | Cases in 1990 | Age-standardized rate in 1990 (per 100 000) | Cases in 2021 | Age-standardized rate in 2021 (per 100 000) | AAPC (95% CI) |
| **Global** | 5055219.9 (4681475.9 to 5465260.6) | 323.9 (288.8 to 360) | 5881661.5 (5453313.4 to 6356782.1) | 310 (276.8 to 344.5) | -0.1404 (-0.1425 to -0.1387) |
| **Sex** |  |  |  |  |  |
| Female | 3146679 (2912748.5 to 3400946.7) | 408.9 (364.4 to 454.2) | 3660315.7 (3399377.8 to 3950478.4) | 394.8 (351.9 to 438.5) | -0.1125 (-0.1143 to -0.1105) |
| Male | 1908540.8 (1764782.1 to 2062619.8) | 241.3 (215.2 to 268.2) | 2221345.8 (2060691 to 2404196.6) | 229 (204.1 to 254.2) | -0.1673 (-0.1700 to -0.1643) |
| **SDI level** |  |  |  |  |  |
| High SDI | 815246.6 (757355 to 874380.4) | 399.6 (358.5 to 442.6) | 739547.1 (687678.9 to 793757) | 384.8 (345.3 to 426.3) | -0.1180 (-0.1292 to -0.1059) |
| High-middle SDI | 969050.5 (896566 to 1051775.9) | 330.1 (293.4 to 366.9) | 747011.7 (692569.7 to 806945.6) | 325.6 (289.9 to 362.6) | -0.0442 (-0.0451 to -0.0432) |
| Middle SDI | 1814210.4 (1679564.2 to 1970134.2) | 325 (289.1 to 361.5) | 1761852.4 (1630216 to 1908142.4) | 316 (281.5 to 351.5) | -0.0897 (-0.0908 to -0.0889) |
| Low-middle SDI | 1021610.7 (948747.1 to 1104131) | 288.7 (257 to 320.2) | 1592107.4 (1479181.6 to 1722268.8) | 286.3 (254.8 to 318.1) | -0.0273 (-0.0277 to -0.0268) |
| Low SDI | 430731.1 (401128.3 to 464193.8) | 287.1 (255.6 to 319.4) | 1036558 (962716.8 to 1117323.9) | 287.6 (256.1 to 319.6) | 0.0049 ( 0.0047 to 0.0052) |
| **GBD Region** |  |  |  |  |  |
| Central Asia | 59983.1 (55539.2 to 65165.2) | 304.5 (268.1 to 342.1) | 66648.5 (61801.5 to 72410.3) | 302.5 (266.4 to 339.9) | -0.0210 (-0.0214 to -0.0205) |
| South Asia | 885022.4 (818352.9 to 955949.8) | 269.1 (238.2 to 300.2) | 1438950 (1327876.8 to 1556899.1) | 269.4 (238.5 to 300.6) | 0.0038 ( 0.0035 to 0.0042) |
| Southeast Asia | 560657 (519162.2 to 605983.3) | 381.5 (339 to 424.8) | 659614.9 (609992.1 to 713561.8) | 379.2 (336.8 to 422.2) | -0.0199 (-0.0202 to -0.0196) |
| East Asia | 1324749.6 (1219233.2 to 1446772.1) | 336.3 (297.2 to 378.3) | 805917 (741446.5 to 875208.2) | 331.9 (293.3 to 373.2) | -0.0434 (-0.0444 to -0.0424) |
| High-income Asia Pacific | 165051.6 (152398.1 to 178072.7) | 380.7 (338.7 to 425.1) | 104172.6 (96169.8 to 112259.7) | 380.6 (338.7 to 425) | -0.0007 (-0.0008 to -0.0005) |
| Western Europe | 320560.9 (296824.1 to 346583.3) | 369.2 (328.9 to 410.5) | 273517.5 (253816.6 to 294599.9) | 369.4 (328.9 to 410.9) | 0.0013 ( 0.0013 to 0.0014) |
| Central Europe | 88317.3 (81903.8 to 95152.1) | 303.5 (269.1 to 340.3) | 55714.5 (51662.3 to 60052.2) | 302.7 (268.5 to 339.4) | -0.0090 (-0.0092 to -0.0088) |
| Eastern Europe | 144883.9 (134313 to 156176) | 304.8 (269.9 to 343) | 98934 (91896 to 106728) | 304 (269.1 to 342.2) | -0.0083 (-0.0085 to -0.0081) |
| Central Sub-Saharan Africa | 49137.4 (45725.5 to 53233.8) | 292.1 (258.3 to 329) | 126967.7 (118115.2 to 137539.5) | 292 (258.2 to 328.9) | -0.0014 (-0.0016 to -0.0011) |
| Eastern Sub-Saharan Africa | 176242.5 (163207 to 189836.6) | 296.2 (262.2 to 329) | 418940.7 (387550.6 to 451891.2) | 294.8 (261.6 to 328.6) | -0.0149 (-0.0161 to -0.0139) |
| Southern Sub-Saharan Africa | 49744.5 (46045.3 to 53775.4) | 295.5 (263 to 330.5) | 63679.5 (58964.5 to 68957.3) | 292.6 (260.7 to 327.1) | -0.0327 (-0.0329 to -0.0325) |
| Western Sub-Saharan Africa | 172063.5 (160022.3 to 185953) | 296.9 (265 to 331.4) | 461612.5 (429140.6 to 498847.5) | 295.7 (264 to 330) | -0.0130 (-0.0136 to -0.0123) |
| North Africa and Middle East | 289575.3 (267832 to 313527.5) | 272.4 (241 to 304.5) | 440778.7 (407354.1 to 477369.3) | 272.4 (240.4 to 304.9) | 0.0000 (-0.0004 to 0.0005) |
| Andean Latin America | 37022.9 (34255.8 to 40172.2) | 306.1 (269.4 to 343.9) | 53322.4 (49318.4 to 58115.7) | 302.2 (266.1 to 339.5) | -0.0419 (-0.0425 to -0.0413) |
| Tropical Latin America | 145362.7 (134824.7 to 156812.4) | 307.2 (271.2 to 346) | 160091.6 (148133.3 to 172536.7) | 305.3 (269.7 to 343.8) | -0.0199 (-0.0201 to -0.0197) |
| High-income North America | 312644.6 (291827.4 to 334003.1) | 489.9 (441.6 to 538.9) | 318339.6 (297896 to 340348.4) | 435.2 (392.3 to 480.3) | -0.3733 (-0.4067 to -0.3346) |
| Caribbean | 33142.6 (30631.8 to 36101.4) | 305.9 (269.3 to 343.7) | 35200 (32542 to 38368.7) | 304.1 (267.7 to 341.7) | -0.0193 (-0.0197 to -0.0189) |
| Central Latin America | 164303.2 (152484.6 to 176815.2) | 307.6 (272.9 to 345.3) | 202164.9 (187685.7 to 217794) | 305.5 (271 to 342.8) | -0.0224 (-0.0227 to -0.0221) |
| Southern Latin America | 50519 (46808.7 to 54497.1) | 383.8 (339.2 to 430) | 60490.5 (56052.6 to 65204.5) | 383.4 (338.7 to 429.3) | -0.0039 (-0.0040 to -0.0038) |
| Oceania | 7240.9 (6672.2 to 7841.5) | 352 (312.3 to 397.5) | 14086.1 (12986.1 to 15273.1) | 350.7 (311.2 to 396.1) | -0.0118 (-0.0123 to -0.0113) |
| Australasia | 18995.2 (17674.5 to 20484.5) | 383.5 (340 to 428.3) | 22518 (20945.4 to 24254.2) | 383.2 (339.9 to 427.8) | -0.0023 (-0.0024 to -0.0022) |

AAPC, Average annual percentage change.

| Table S2 Age-standardized DALYs and AAPC of alopecia areata in adolescents and young adults aged 10-24 years at global and regional level, 1990-2021 | | | | | |
| --- | --- | --- | --- | --- | --- |
|  | DALYs (95% UI) |  |  |  |  |
|  | Cases in 1990 | Age-standardized rate in 1990 (per 100 000) | Cases in 2021 | Age-standardized rate in 2021 (per 100 000) | AAPC (95% CI) |
| **Global** | 90353 (57820.5 to 129223.3) | 5.8 (3.7 to 8.4) | 105334.5 (67557.2 to 150574.7) | 5.6 (3.6 to 8.1) | -0.1351 (-0.1382 to -0.1317) |
| **Sex** |  |  |  |  |  |
| Female | 56170 (35744.8 to 80207) | 7.3 (4.6 to 10.7) | 65409.7 (41650.4 to 93511.7) | 7.1 (4.5 to 10.3) | -0.1085 (-0.1122 to -0.1046) |
| Male | 34183 (21895.1 to 49247.3) | 4.3 (2.8 to 6.3) | 39924.8 (25764 to 57248.1) | 4.1 (2.6 to 6) | -0.1568 (-0.1644 to -0.1494) |
| **SDI level** |  |  |  |  |  |
| High SDI | 14507.5 (9310.1 to 20891.5) | 7.1 (4.5 to 10.4) | 13180 (8430.9 to 18861.4) | 6.9 (4.4 to 10) | -0.1144 (-0.1268 to -0.1017) |
| High-middle SDI | 17408.6 (10982.1 to 25229.2) | 5.9 (3.7 to 8.7) | 13433.1 (8585.7 to 19455.4) | 5.9 (3.7 to 8.6) | -0.0404 (-0.0458 to -0.0361) |
| Middle SDI | 32518.1 (20771.5 to 47265.9) | 5.8 (3.7 to 8.5) | 31622.7 (20169.4 to 45475.4) | 5.7 (3.6 to 8.2) | -0.0858 (-0.0881 to -0.0835) |
| Low-middle SDI | 18192 (11656.4 to 26242.8) | 5.1 (3.3 to 7.5) | 28520.2 (18223.8 to 40868.2) | 5.1 (3.3 to 7.4) | -0.0100 (-0.0146 to -0.0038) |
| Low SDI | 7648.6 (4871.1 to 10811.2) | 5.1 (3.2 to 7.3) | 18496.5 (11981.2 to 26559.9) | 5.1 (3.3 to 7.5) | 0.0266 ( 0.0224 to 0.0307) |
| **GBD Region** |  |  |  |  |  |
| Central Asia | 1072.5 (665.9 to 1555) | 5.4 (3.4 to 8.2) | 1196.3 (765 to 1721.3) | 5.4 (3.4 to 8.1) | -0.0171 (-0.0239 to -0.0102) |
| South Asia | 15734.1 (10035.8 to 22686.4) | 4.8 (3 to 7) | 25782.5 (16649.3 to 36958.3) | 4.8 (3.1 to 7) | 0.0226 ( 0.0176 to 0.0305) |
| Southeast Asia | 10039.1 (6302.6 to 14380.2) | 6.8 (4.3 to 10) | 11841.2 (7448.2 to 17118.2) | 6.8 (4.3 to 10) | -0.0099 (-0.0144 to -0.0054) |
| East Asia | 23828.4 (15138.6 to 34630.9) | 6 (3.8 to 8.9) | 14541.1 (9218.2 to 21118.6) | 6 (3.8 to 8.8) | -0.0312 (-0.0372 to -0.0256) |
| High-income Asia Pacific | 2958.7 (1916.4 to 4226.9) | 6.8 (4.3 to 10) | 1864.8 (1164.2 to 2691.3) | 6.8 (4.3 to 10.1) | -0.0056 (-0.0120 to 0.0008) |
| Western Europe | 5706.1 (3615.3 to 8294.1) | 6.6 (4.1 to 9.7) | 4866.8 (3116.6 to 7126.6) | 6.6 (4.2 to 9.7) | 0.0047 (-0.0011 to 0.0102) |
| Central Europe | 1585.7 (1014.8 to 2306.1) | 5.4 (3.4 to 8.1) | 998.8 (641.7 to 1450.1) | 5.4 (3.4 to 8) | -0.0115 (-0.0191 to -0.0056) |
| Eastern Europe | 2605.1 (1662.1 to 3773.9) | 5.5 (3.5 to 8.2) | 1775.8 (1149.3 to 2561.2) | 5.5 (3.5 to 8.1) | -0.0060 (-0.0104 to -0.0016) |
| Central Sub-Saharan Africa | 869.6 (535.6 to 1279.8) | 5.2 (3.1 to 7.9) | 2256.7 (1405.1 to 3371) | 5.2 (3.1 to 8.2) | 0.0152 (-0.0064 to 0.0444) |
| Eastern Sub-Saharan Africa | 3132.4 (1989.9 to 4462.8) | 5.3 (3.3 to 7.6) | 7495.2 (4888.2 to 10816.3) | 5.3 (3.3 to 7.7) | 0.0073 (-0.0007 to 0.0145) |
| Southern Sub-Saharan Africa | 890.2 (575.7 to 1293.9) | 5.3 (3.3 to 7.8) | 1138.9 (722 to 1648.3) | 5.2 (3.2 to 7.8) | -0.0313 (-0.0380 to -0.0245) |
| Western Sub-Saharan Africa | 3058.7 (1932.4 to 4370.5) | 5.3 (3.3 to 7.8) | 8236.6 (5340.3 to 11750) | 5.3 (3.4 to 7.7) | -0.0046 (-0.0091 to 0.0001) |
| North Africa and Middle East | 5185.9 (3252 to 7557.2) | 4.9 (3 to 7.2) | 7895.4 (4945.1 to 11382.1) | 4.9 (3.1 to 7.3) | 0.0038 (-0.0040 to 0.0117) |
| Andean Latin America | 665 (417.6 to 997.6) | 5.5 (3.3 to 8.4) | 954.1 (588.4 to 1405.3) | 5.4 (3.3 to 8.3) | -0.0456 (-0.0734 to -0.0199) |
| Tropical Latin America | 2592.4 (1665.3 to 3757.6) | 5.5 (3.5 to 8) | 2858.6 (1803.7 to 4152.5) | 5.4 (3.4 to 8.1) | -0.0101 (-0.0175 to -0.0027) |
| High-income North America | 5522.7 (3554.4 to 7989.1) | 8.7 (5.5 to 12.7) | 5658.2 (3666.7 to 8156.8) | 7.7 (5 to 11.4) | -0.3589 (-0.3885 to -0.3259) |
| Caribbean | 592.2 (373.8 to 863.6) | 5.5 (3.4 to 8.2) | 627.6 (392 to 908.9) | 5.4 (3.3 to 8.1) | -0.0146 (-0.0219 to -0.0073) |
| Central Latin America | 2945 (1872.2 to 4223.7) | 5.5 (3.4 to 8) | 3620.1 (2303.6 to 5198.5) | 5.5 (3.4 to 8.1) | -0.0226 (-0.0272 to -0.0180) |
| Southern Latin America | 903.2 (561.3 to 1317.5) | 6.9 (4.1 to 10.6) | 1075.2 (672.2 to 1568.2) | 6.8 (4.1 to 10.4) | -0.0176 (-0.0403 to 0.0005) |
| Oceania | 128.9 (80.3 to 191.3) | 6.3 (3.7 to 9.5) | 251.3 (156.5 to 369.9) | 6.3 (3.6 to 9.6) | -0.0020 (-0.0165 to 0.0123) |
| Australasia | 337.2 (207.9 to 487.5) | 6.8 (4 to 10.6) | 399.1 (244.7 to 583.8) | 6.8 (4 to 10.5) | -0.0016 (-0.0156 to 0.0126) |

AAPC, Average annual percentage change; DALYs, disability adjusted life-years.

| Table S3 Age-specific prevalence, incidence, and DALYs of alopecia areata in adolescents and young adults aged 10-24 years at global level, 1990-2021 | | | | | |
| --- | --- | --- | --- | --- | --- |
| Age group | Cases in 1990 | Age-specific rate in 1990 (per 100 000) | Cases in 2021 | Age-specific rate in 2021 (per 100 000) | AAPC (95% CI) |
|  | Prevalence (95% UI) |  |  |  |  |
| 10-14 years | 525650 (473194.7 to 576923) | 98.1 (88.3 to 107.7) | 638665.3 (575789.4 to 701975.7) | 95.8 (86.4 to 105.3) | -0.0764 (-0.0778 to -0.0748) |
| 15-19 years | 879236.9 (791428 to 973570) | 169.3 (152.4 to 187.4) | 1011782.6 (912706.9 to 1116255.9) | 162.1 (146.3 to 178.9) | -0.1384 (-0.1403 to -0.1364) |
| 20-24 years | 1307552.3 (1176115.7 to 1448652) | 265.7 (239 to 294.4) | 1508398.2 (1355274.1 to 1666327.9) | 252.6 (227 to 279) | -0.1628 (-0.1660 to -0.1597) |
|  | Incidence (95% UI) |  |  |  |  |
| 10-14 years | 994915.8 (879502.7 to 1112717.2) | 185.7 (164.2 to 207.7) | 1205973.8 (1067136.8 to 1348537.5) | 180.9 (160.1 to 202.3) | -0.0846 (-0.0864 to -0.0827) |
| 15-19 years | 1629351.9 (1442491.1 to 1813285.1) | 313.7 (277.7 to 349.1) | 1875869.1 (1667283.9 to 2085476.2) | 300.6 (267.2 to 334.2) | -0.1374 (-0.1394 to -0.1354) |
| 20-24 years | 2430952.2 (2186042.4 to 2692183.5) | 494 (444.2 to 547.1) | 2799818.5 (2516922.4 to 3102142.9) | 468.9 (421.5 to 519.5) | -0.1691 (-0.1714 to -0.1667) |
|  | DALYs (95% UI) |  |  |  |  |
| 10-14 years | 17629.6 (11479 to 25755.5) | 3.3 (2.1 to 4.8) | 21395.7 (14049.3 to 31110) | 3.2 (2.1 to 4.7) | -0.0794 (-0.0846 to -0.0745) |
| 15-19 years | 29305.2 (18720 to 42739.7) | 5.6 (3.6 to 8.2) | 33792.6 (21971.7 to 48970.8) | 5.4 (3.5 to 7.8) | -0.1303 (-0.1352 to -0.1256) |
| 20-24 years | 43418.2 (27468.2 to 62867.6) | 8.8 (5.6 to 12.8) | 50146.3 (31725.1 to 73276.1) | 8.4 (5.3 to 12.3) | -0.1614 (-0.1646 to -0.1582) |

AAPC, Average annual percentage change; DALYs, disability adjusted life-years.

| Table S4 Age-standardized prevalence, incidence, and DALYs of alopecia areata in adolescents and young adults aged 10-24 years in 2021 and their AAPCs between 1990-2021 in 204 countries and territories. | | | | | | | |
| --- | --- | --- | --- | --- | --- | --- | --- |
|  | **Age-standardized rate in 2021 (per 100 000)** | | |  | **AAPC 1990-2021** | | |
|  | **Incidence (95% UI)** | **Prevalence (95% UI)** | **DALYs (95% UI)** |  | **Incidence (95% CI)** | **Prevalence (95% CI)** | **DALYs (95% CI)** |
|  |  |  |  |  |  |  |  |
| Afghanistan | 272.6 (239.9 to 305) | 146.7 (130.6 to 163.8) | 4.9 (2.7 to 7.9) |  | -0.0729 (-0.0776 to -0.0673) | -0.0728 (-0.0774 to -0.0672) | -0.0543 (-0.0846 to -0.0120) |
| Albania | 300.1 (264.2 to 337.2) | 160.9 (143.2 to 179.4) | 5.4 (3 to 8.5) |  | -0.0435 (-0.0446 to -0.0424) | -0.0437 (-0.0448 to -0.0425) | -0.0162 (-0.0432 to 0.0124) |
| Algeria | 273.2 (240.3 to 305.6) | 147 (130.8 to 164.1) | 4.9 (2.7 to 7.8) |  | -0.0076 (-0.0079 to -0.0074) | -0.0077 (-0.0079 to -0.0074) | -0.0059 (-0.0241 to 0.0131) |
| American Samoa | 352.8 (313 to 398.4) | 189.1 (169.1 to 211.5) | 6.4 (3.5 to 10.1) |  | -0.0095 (-0.0102 to -0.0088) | -0.0097 (-0.0104 to -0.0090) | 0.0246 ( 0.0073 to 0.0420) |
| Andorra | 368.9 (326.3 to 412.3) | 196.8 (175.6 to 219.1) | 6.6 (3.7 to 10.6) |  | -0.0029 (-0.0040 to -0.0019) | -0.0022 (-0.0032 to -0.0014) | 0.0051 (-0.0055 to 0.0161) |
| Angola | 294.7 (260.4 to 332.1) | 158.3 (141.4 to 176.5) | 5.2 (3 to 8.4) |  | 0.0317 ( 0.0308 to 0.0326) | 0.0317 ( 0.0308 to 0.0327) | 0.0335 ( 0.0101 to 0.0543) |
| Antigua and Barbuda | 303.3 (267 to 340.8) | 162.7 (144.7 to 181.4) | 5.4 (3 to 8.6) |  | -0.0258 (-0.0270 to -0.0243) | -0.0259 (-0.0271 to -0.0244) | -0.0282 (-0.0422 to -0.0128) |
| Argentina | 383.5 (338.9 to 429.5) | 204.8 (182.3 to 227.9) | 6.8 (4 to 10.6) |  | -0.0035 (-0.0036 to -0.0034) | -0.0038 (-0.0039 to -0.0037) | 0.0046 (-0.0096 to 0.0192) |
| Armenia | 300 (264.2 to 337.1) | 160.9 (143.1 to 179.4) | 5.4 (3.1 to 8.6) |  | -0.0424 (-0.0434 to -0.0415) | -0.0425 (-0.0435 to -0.0415) | -0.0333 (-0.0534 to -0.0128) |
| Australia | 383 (338.4 to 428.8) | 204.6 (182 to 227.5) | 6.8 (3.9 to 10.7) |  | -0.0018 (-0.0018 to -0.0017) | -0.0021 (-0.0022 to -0.0021) | -0.0011 (-0.0186 to 0.0161) |
| Austria | 369 (326.4 to 412.5) | 196.9 (175.7 to 219.2) | 6.5 (3.7 to 10.4) |  | 0.0026 ( 0.0023 to 0.0028) | 0.0019 ( 0.0017 to 0.0020) | 0.0098 (-0.0036 to 0.0236) |
| Azerbaijan | 300.1 (264.3 to 337.2) | 161 (143.2 to 179.5) | 5.4 (3.1 to 8.5) |  | -0.0482 (-0.0492 to -0.0472) | -0.0482 (-0.0494 to -0.0472) | -0.0303 (-0.0484 to -0.0121) |
| Bahamas | 305.7 (269.1 to 343.5) | 164 (145.8 to 183) | 5.5 (3.1 to 8.8) |  | 0.0055 ( 0.0051 to 0.0059) | 0.0055 ( 0.0051 to 0.0059) | 0.0117 (-0.0100 to 0.0336) |
| Bahrain | 261.4 (230.3 to 292.4) | 140.6 (125.2 to 156.9) | 4.7 (2.5 to 7.7) |  | -0.1251 (-0.1402 to -0.1125) | -0.1252 (-0.1402 to -0.1124) | -0.1231 (-0.1546 to -0.0916) |
| Bangladesh | 273.8 (242.5 to 308.8) | 147 (131.1 to 164.1) | 4.9 (2.7 to 7.9) |  | 0.0170 ( 0.0158 to 0.0181) | 0.0171 ( 0.0159 to 0.0182) | 0.0268 ( 0.0044 to 0.0491) |
| Barbados | 303.5 (267.3 to 341.1) | 162.8 (144.8 to 181.6) | 5.4 (3 to 8.5) |  | -0.0072 (-0.0076 to -0.0069) | -0.0072 (-0.0076 to -0.0069) | 0.0015 (-0.0137 to 0.0165) |
| Belarus | 302.2 (266.1 to 339.5) | 162.1 (144.2 to 180.7) | 5.4 (3.1 to 8.3) |  | -0.0286 (-0.0290 to -0.0282) | -0.0287 (-0.0291 to -0.0284) | -0.0451 (-0.0662 to -0.0187) |
| Belgium | 368.7 (326.1 to 412) | 196.7 (175.5 to 219) | 6.5 (3.7 to 10.2) |  | -0.0006 (-0.0007 to -0.0005) | -0.0005 (-0.0006 to -0.0004) | -0.0218 (-0.0357 to -0.0076) |
| Belize | 305.8 (269.2 to 343.6) | 164 (145.9 to 183) | 5.5 (3.1 to 8.6) |  | 0.0074 ( 0.0071 to 0.0077) | 0.0074 ( 0.0071 to 0.0077) | 0.0017 (-0.0182 to 0.0220) |
| Benin | 294.8 (260.5 to 332.3) | 158.4 (141.5 to 176.6) | 5.3 (3 to 8.5) |  | -0.0310 (-0.0316 to -0.0303) | -0.0312 (-0.0318 to -0.0305) | -0.0221 (-0.0436 to -0.0007) |
| Bermuda | 305 (268.5 to 342.7) | 163.6 (145.5 to 182.6) | 5.5 (3 to 8.6) |  | 0.0103 ( 0.0100 to 0.0107) | 0.0104 ( 0.0100 to 0.0108) | 0.0167 (-0.0037 to 0.0349) |
| Bhutan | 268.3 (237.6 to 302.2) | 144.1 (128.5 to 160.8) | 4.8 (2.6 to 7.7) |  | 0.0660 ( 0.0620 to 0.0700) | 0.0660 ( 0.0621 to 0.0700) | 0.0589 ( 0.0379 to 0.0799) |
| Bolivia (Plurinational State of) | 304.1 (267.7 to 341.7) | 163.1 (145.1 to 181.9) | 5.4 (3 to 8.6) |  | -0.0229 (-0.0232 to -0.0226) | -0.0229 (-0.0232 to -0.0226) | -0.0176 (-0.0415 to 0.0067) |
| Bosnia and Herzegovina | 302.7 (266.5 to 340.1) | 162.4 (144.4 to 181.1) | 5.4 (3 to 8.6) |  | 0.0128 ( 0.0097 to 0.0167) | 0.0129 ( 0.0098 to 0.0168) | 0.0079 (-0.0725 to 0.0691) |
| Botswana | 292.2 (258.3 to 329.1) | 157 (140.2 to 175) | 5.2 (2.8 to 8.4) |  | -0.0510 (-0.0514 to -0.0508) | -0.0510 (-0.0514 to -0.0508) | -0.0237 (-0.0439 to -0.0033) |
| Brazil | 305.4 (269.5 to 344.2) | 164.1 (146 to 183.6) | 5.5 (3.4 to 8.1) |  | -0.0195 (-0.0197 to -0.0193) | -0.0196 (-0.0198 to -0.0193) | -0.0097 (-0.0172 to -0.0022) |
| Brunei Darussalam | 378.6 (334.2 to 424) | 202.2 (180.1 to 225.3) | 6.8 (3.8 to 10.6) |  | -0.0086 (-0.0090 to -0.0083) | -0.0094 (-0.0097 to -0.0092) | -0.0043 (-0.0162 to 0.0077) |
| Bulgaria | 302.1 (266 to 339.5) | 162.1 (144.2 to 180.7) | 5.4 (3 to 8.6) |  | -0.0098 (-0.0102 to -0.0095) | -0.0099 (-0.0103 to -0.0095) | -0.0079 (-0.0287 to 0.0133) |
| Burkina Faso | 297.4 (262.7 to 335.3) | 159.8 (142.8 to 178.2) | 5.3 (2.9 to 8.6) |  | 0.0118 ( 0.0115 to 0.0122) | 0.0116 ( 0.0110 to 0.0121) | 0.0450 ( 0.0273 to 0.0627) |
| Burundi | 296 (261.6 to 331.5) | 159.1 (141.4 to 177.8) | 5.3 (3 to 8.5) |  | -0.0004 (-0.0045 to 0.0039) | -0.0005 (-0.0046 to 0.0038) | 0.0161 ( 0.0014 to 0.0310) |
| Cabo Verde | 291.1 (257.5 to 327.8) | 156.4 (139.6 to 174.4) | 5.2 (2.9 to 8.4) |  | -0.0236 (-0.0237 to -0.0234) | -0.0236 (-0.0238 to -0.0235) | -0.0120 (-0.0331 to 0.0054) |
| Cambodia | 378.3 (334.4 to 425.9) | 202.9 (180.3 to 227.1) | 6.8 (3.8 to 10.8) |  | -0.0372 (-0.0376 to -0.0367) | -0.0372 (-0.0376 to -0.0367) | -0.0252 (-0.0404 to -0.0102) |
| Cameroon | 293 (259 to 330.1) | 157.4 (140.6 to 175.5) | 5.2 (2.9 to 8.4) |  | -0.0352 (-0.0355 to -0.0349) | -0.0353 (-0.0356 to -0.0350) | -0.0400 (-0.0694 to -0.0177) |
| Canada | 426.1 (375.3 to 478.8) | 227.5 (201.8 to 253.5) | 7.6 (4.3 to 11.8) |  | -0.0103 (-0.0105 to -0.0101) | -0.0108 (-0.0110 to -0.0107) | -0.0121 (-0.0257 to 0.0016) |
| Central African Republic | 294.5 (260.2 to 331.9) | 158.2 (141.3 to 176.4) | 5.2 (2.9 to 8.4) |  | 0.0026 ( 0.0015 to 0.0038) | 0.0026 ( 0.0014 to 0.0038) | 0.0200 ( 0.0043 to 0.0358) |
| Chad | 294.4 (260.1 to 331.7) | 158.1 (141.3 to 176.3) | 5.2 (2.9 to 8.4) |  | -0.0147 (-0.0151 to -0.0143) | -0.0147 (-0.0151 to -0.0143) | -0.0172 (-0.0332 to -0.0010) |
| Chile | 383 (338.4 to 428.9) | 204.6 (182 to 227.5) | 6.8 (3.9 to 10.7) |  | -0.0058 (-0.0060 to -0.0056) | -0.0067 (-0.0070 to -0.0066) | -0.0358 (-0.0623 to -0.0158) |
| China | 331.7 (292.9 to 373.5) | 178 (158.1 to 199.9) | 6 (3.8 to 8.8) |  | -0.0433 (-0.0443 to -0.0423) | -0.0432 (-0.0442 to -0.0423) | -0.0301 (-0.0360 to -0.0248) |
| Colombia | 302.7 (266.5 to 340.1) | 162.4 (144.4 to 181.1) | 5.4 (3 to 8.7) |  | -0.0482 (-0.0492 to -0.0471) | -0.0484 (-0.0494 to -0.0473) | -0.0358 (-0.0604 to -0.0106) |
| Comoros | 293.1 (259.1 to 328.2) | 157.6 (140 to 175.9) | 5.3 (2.9 to 8.3) |  | -0.0107 (-0.0108 to -0.0106) | -0.0107 (-0.0108 to -0.0106) | 0.0024 (-0.0113 to 0.0156) |
| Congo | 293.6 (259.4 to 330.8) | 157.7 (140.9 to 175.8) | 5.2 (3 to 8.7) |  | 0.0019 ( 0.0011 to 0.0028) | 0.0020 ( 0.0011 to 0.0029) | 0.0173 (-0.0081 to 0.0428) |
| Cook Islands | 359.7 (318.9 to 406.1) | 192.8 (172.3 to 215.8) | 6.5 (3.5 to 10.5) |  | 0.0664 ( 0.0635 to 0.0694) | 0.0667 ( 0.0637 to 0.0699) | 0.0710 ( 0.0522 to 0.0905) |
| Costa Rica | 305.3 (268.8 to 343) | 163.8 (145.7 to 182.7) | 5.5 (3 to 8.8) |  | 0.0042 ( 0.0039 to 0.0045) | 0.0042 ( 0.0039 to 0.0045) | 0.0076 (-0.0122 to 0.0276) |
| Côte d'Ivoire | 290 (256.5 to 326.4) | 155.8 (139.1 to 173.7) | 5.2 (2.9 to 8.1) |  | -0.0392 (-0.0401 to -0.0384) | -0.0391 (-0.0401 to -0.0384) | -0.0086 (-0.0260 to 0.0092) |
| Croatia | 302.6 (266.4 to 340) | 162.3 (144.4 to 181) | 5.4 (3.1 to 8.9) |  | -0.0061 (-0.0066 to -0.0056) | -0.0066 (-0.0072 to -0.0061) | -0.0107 (-0.0252 to 0.0041) |
| Cuba | 301.8 (265.8 to 339.2) | 161.9 (144 to 180.5) | 5.4 (3 to 8.6) |  | -0.0125 (-0.0126 to -0.0123) | -0.0125 (-0.0127 to -0.0123) | -0.0031 (-0.0179 to 0.0240) |
| Cyprus | 368.8 (326.2 to 412.2) | 196.8 (175.6 to 219.1) | 6.6 (3.7 to 10.3) |  | -0.0008 (-0.0010 to -0.0006) | -0.0007 (-0.0009 to -0.0005) | 0.0100 (-0.0049 to 0.0247) |
| Czechia | 302.6 (266.4 to 340) | 162.3 (144.4 to 181) | 5.4 (3 to 8.7) |  | -0.0041 (-0.0043 to -0.0039) | -0.0041 (-0.0045 to -0.0038) | -0.0046 (-0.0221 to 0.0133) |
| Democratic People's Republic of Korea | 335.7 (297.1 to 376) | 179.8 (160.5 to 200.7) | 6.1 (3.4 to 9.5) |  | -0.1146 (-0.1159 to -0.1131) | -0.1149 (-0.1162 to -0.1133) | -0.1159 (-0.1323 to -0.0994) |
| Democratic Republic of the Congo | 291.1 (257.4 to 327.7) | 156.4 (139.6 to 174.3) | 5.2 (2.9 to 8.3) |  | -0.0092 (-0.0094 to -0.0090) | -0.0092 (-0.0094 to -0.0090) | 0.0344 ( 0.0163 to 0.0527) |
| Denmark | 368.8 (326.2 to 412.1) | 196.8 (175.6 to 219.1) | 6.6 (3.8 to 10.4) |  | -0.0007 (-0.0010 to -0.0005) | -0.0006 (-0.0009 to -0.0004) | -0.0060 (-0.0216 to 0.0092) |
| Djibouti | 284.4 (251.4 to 318.5) | 152.9 (136.1 to 170.4) | 5.1 (2.7 to 8.3) |  | -0.0531 (-0.0581 to -0.0473) | -0.0530 (-0.0581 to -0.0470) | -0.0479 (-0.0910 to -0.0047) |
| Dominica | 302 (265.9 to 339.3) | 162 (144.1 to 180.6) | 5.4 (3 to 8.6) |  | 0.0008 (-0.0006 to 0.0024) | 0.0007 (-0.0007 to 0.0024) | 0.0158 (-0.0071 to 0.0393) |
| Dominican Republic | 304.4 (268 to 342) | 163.3 (145.2 to 182.1) | 5.4 (3.1 to 8.6) |  | -0.0423 (-0.0430 to -0.0416) | -0.0424 (-0.0431 to -0.0417) | -0.0434 (-0.0621 to -0.0254) |
| Ecuador | 303.9 (267.6 to 341.5) | 163 (145 to 181.8) | 5.4 (3.1 to 8.7) |  | -0.0269 (-0.0274 to -0.0263) | -0.0270 (-0.0275 to -0.0265) | -0.0416 (-0.0761 to -0.0068) |
| Egypt | 272.4 (238.3 to 305.9) | 146.7 (130 to 164.2) | 4.9 (2.7 to 7.9) |  | 0.0102 ( 0.0097 to 0.0107) | 0.0137 ( 0.0130 to 0.0143) | 0.0367 ( 0.0075 to 0.0663) |
| El Salvador | 305.4 (268.8 to 343.1) | 163.8 (145.7 to 182.8) | 5.5 (3 to 8.7) |  | -0.0264 (-0.0267 to -0.0261) | -0.0264 (-0.0267 to -0.0261) | -0.0123 (-0.0367 to 0.0123) |
| Equatorial Guinea | 277.9 (246.4 to 312.4) | 149.3 (133.3 to 166.4) | 4.9 (2.8 to 8) |  | -0.2072 (-0.2087 to -0.2058) | -0.2071 (-0.2086 to -0.2056) | -0.1649 (-0.1870 to -0.1390) |
| Eritrea | 290.4 (256.7 to 325.1) | 156.1 (138.8 to 174.2) | 5.2 (2.9 to 8.4) |  | -0.0211 (-0.0221 to -0.0200) | -0.0214 (-0.0223 to -0.0206) | 0.0404 ( 0.0185 to 0.0622) |
| Estonia | 302.5 (266.4 to 340) | 162.3 (144.4 to 181) | 5.4 (3 to 8.7) |  | 0.0089 ( 0.0072 to 0.0109) | 0.0090 ( 0.0073 to 0.0110) | 0.0031 (-0.0165 to 0.0230) |
| Eswatini | 291.8 (258 to 328.7) | 156.8 (140 to 174.8) | 5.2 (2.9 to 8.2) |  | -0.0667 (-0.0678 to -0.0657) | -0.0667 (-0.0678 to -0.0657) | -0.0810 (-0.1224 to -0.0420) |
| Ethiopia | 293.3 (258.9 to 328.6) | 157.8 (140.6 to 176.1) | 5.3 (3.2 to 7.9) |  | -0.0264 (-0.0272 to -0.0256) | -0.0266 (-0.0274 to -0.0258) | 0.0060 (-0.0041 to 0.0162) |
| Fiji | 352.9 (313.1 to 398.6) | 189.2 (169.2 to 211.6) | 6.4 (3.5 to 10.2) |  | -0.0057 (-0.0060 to -0.0054) | -0.0058 (-0.0060 to -0.0055) | 0.0165 ( 0.0028 to 0.0298) |
| Finland | 368.9 (326.3 to 412.3) | 196.8 (175.6 to 219.1) | 6.5 (3.7 to 10.4) |  | 0.0012 ( 0.0010 to 0.0013) | 0.0010 ( 0.0008 to 0.0011) | 0.0089 (-0.0111 to 0.0294) |
| France | 368.7 (326.2 to 412.1) | 196.7 (175.6 to 219.1) | 6.6 (3.8 to 10.5) |  | 0.0010 ( 0.0010 to 0.0011) | 0.0008 ( 0.0008 to 0.0009) | -0.0039 (-0.0185 to 0.0108) |
| Gabon | 297.9 (263.1 to 335.9) | 160 (143 to 178.5) | 5.3 (3 to 8.5) |  | 0.0349 ( 0.0342 to 0.0357) | 0.0350 ( 0.0344 to 0.0356) | 0.0422 ( 0.0239 to 0.0612) |
| Gambia | 294.6 (260.3 to 332) | 158.3 (141.4 to 176.5) | 5.3 (3 to 8.4) |  | -0.0072 (-0.0075 to -0.0069) | -0.0073 (-0.0076 to -0.0069) | 0.0115 (-0.0047 to 0.0277) |
| Georgia | 299.2 (263.5 to 336.2) | 160.5 (142.8 to 178.9) | 5.4 (2.9 to 8.7) |  | -0.0534 (-0.0540 to -0.0527) | -0.0535 (-0.0542 to -0.0528) | -0.0477 (-0.0647 to -0.0296) |
| Germany | 369.4 (326.7 to 412.9) | 197 (175.8 to 219.4) | 6.6 (3.7 to 10.2) |  | 0.0052 ( 0.0050 to 0.0054) | 0.0039 ( 0.0038 to 0.0041) | 0.0076 (-0.0175 to 0.0320) |
| Ghana | 293.1 (259.1 to 330.3) | 157.5 (140.7 to 175.6) | 5.2 (2.9 to 8.5) |  | -0.0073 (-0.0076 to -0.0070) | -0.0074 (-0.0077 to -0.0072) | -0.0095 (-0.0347 to 0.0230) |
| Greece | 368.6 (326.1 to 412) | 196.7 (175.5 to 219) | 6.5 (3.8 to 10.2) |  | 0.0006 ( 0.0005 to 0.0008) | 0.0005 ( 0.0004 to 0.0006) | 0.0055 (-0.0111 to 0.0225) |
| Greenland | 427.8 (376.7 to 480.7) | 228.5 (202.7 to 254.5) | 7.6 (4.4 to 12.1) |  | 0.0246 ( 0.0206 to 0.0284) | 0.0264 ( 0.0218 to 0.0305) | 0.0141 (-0.0070 to 0.0343) |
| Grenada | 299.8 (264.1 to 336.9) | 160.8 (143.1 to 179.2) | 5.4 (3.1 to 8.4) |  | -0.0411 (-0.0423 to -0.0398) | -0.0413 (-0.0425 to -0.0401) | -0.0333 (-0.0498 to -0.0162) |
| Guam | 350.7 (311.1 to 396.1) | 187.9 (168.1 to 210.2) | 6.3 (3.5 to 10.1) |  | 0.0539 ( 0.0526 to 0.0557) | 0.0543 ( 0.0530 to 0.0561) | 0.0625 ( 0.0426 to 0.0806) |
| Guatemala | 306.3 (269.6 to 344.1) | 164.3 (146.1 to 183.4) | 5.4 (3 to 8.5) |  | -0.0243 (-0.0246 to -0.0240) | -0.0244 (-0.0247 to -0.0241) | -0.0301 (-0.0773 to 0.0293) |
| Guinea-Bissau | 293.4 (259.3 to 330.6) | 157.6 (140.8 to 175.8) | 5.2 (2.9 to 8.4) |  | -0.0213 (-0.0218 to -0.0210) | -0.0213 (-0.0218 to -0.0210) | -0.0195 (-0.0455 to 0.0189) |
| Guinea | 296.1 (261.5 to 333.7) | 159.1 (142.1 to 177.4) | 5.3 (2.9 to 8.7) |  | -0.0038 (-0.0054 to -0.0020) | -0.0039 (-0.0055 to -0.0021) | 0.0222 (-0.0085 to 0.0487) |
| Guyana | 304.3 (267.9 to 341.9) | 163.2 (145.2 to 182.1) | 5.4 (3 to 8.5) |  | -0.0243 (-0.0260 to -0.0229) | -0.0244 (-0.0260 to -0.0229) | 0.0032 (-0.0090 to 0.0155) |
| Haiti | 305.4 (268.8 to 343.1) | 163.8 (145.7 to 182.8) | 5.4 (2.9 to 8.6) |  | -0.0383 (-0.0392 to -0.0372) | -0.0384 (-0.0393 to -0.0373) | -0.0496 (-0.0709 to -0.0192) |
| Honduras | 305.9 (269.3 to 343.7) | 164.1 (145.9 to 183.1) | 5.5 (3 to 8.8) |  | -0.0144 (-0.0148 to -0.0141) | -0.0145 (-0.0148 to -0.0141) | 0.0007 (-0.0184 to 0.0202) |
| Hungary | 302.3 (266.2 to 339.7) | 162.2 (144.3 to 180.8) | 5.4 (3 to 8.7) |  | -0.0048 (-0.0050 to -0.0045) | -0.0048 (-0.0050 to -0.0045) | -0.0189 (-0.0430 to 0.0123) |
| Iceland | 368.7 (326.2 to 412.1) | 196.8 (175.6 to 219.1) | 6.6 (3.6 to 10.1) |  | 0.0008 (-0.0001 to 0.0018) | 0.0007 ( 0.0000 to 0.0015) | 0.0060 (-0.0217 to 0.0280) |
| India | 268.8 (237.6 to 300.6) | 144.6 (128.8 to 161.1) | 4.8 (3.1 to 7) |  | 0.0015 ( 0.0011 to 0.0019) | 0.0017 ( 0.0012 to 0.0021) | 0.0202 ( 0.0158 to 0.0264) |
| Indonesia | 379.4 (334.1 to 425.4) | 203.7 (182.6 to 227.4) | 6.8 (4.2 to 10.1) |  | -0.0280 (-0.0282 to -0.0279) | -0.0281 (-0.0283 to -0.0280) | -0.0197 (-0.0277 to -0.0119) |
| Iran (Islamic Republic of) | 274.1 (241.4 to 308.8) | 147.7 (131.4 to 165.2) | 4.9 (3 to 7.3) |  | -0.0101 (-0.0114 to -0.0088) | -0.0101 (-0.0114 to -0.0088) | -0.0062 (-0.0126 to 0.0002) |
| Iraq | 285.3 (252.6 to 318.6) | 153.6 (137.3 to 171.2) | 5.1 (2.8 to 8.2) |  | 0.0058 ( 0.0045 to 0.0071) | 0.0058 ( 0.0045 to 0.0071) | 0.0223 (-0.0016 to 0.0464) |
| Ireland | 368.7 (326.1 to 412) | 196.7 (175.5 to 219) | 6.6 (3.8 to 10.3) |  | -0.0006 (-0.0009 to -0.0003) | -0.0004 (-0.0007 to -0.0002) | 0.0144 ( 0.0036 to 0.0270) |
| Israel | 368.7 (326.2 to 412.1) | 196.7 (175.6 to 219.1) | 6.6 (3.8 to 10.4) |  | 0.0009 ( 0.0008 to 0.0010) | 0.0008 ( 0.0007 to 0.0009) | 0.0003 (-0.0150 to 0.0159) |
| Italy | 370.8 (327.3 to 417) | 198.2 (177.1 to 222.4) | 6.6 (4.1 to 9.6) |  | 0.0028 ( 0.0027 to 0.0029) | 0.0021 ( 0.0020 to 0.0022) | 0.0047 (-0.0012 to 0.0098) |
| Jamaica | 303.4 (267.1 to 340.9) | 162.7 (144.7 to 181.5) | 5.4 (3 to 8.7) |  | -0.0314 (-0.0316 to -0.0311) | -0.0315 (-0.0318 to -0.0313) | -0.0348 (-0.0581 to -0.0160) |
| Japan | 381.2 (336.8 to 426.2) | 204.1 (182.2 to 227.7) | 6.8 (4.4 to 10) |  | -0.0007 (-0.0008 to -0.0007) | -0.0008 (-0.0009 to -0.0008) | -0.0034 (-0.0081 to 0.0016) |
| Jordan | 270.1 (237.6 to 302.1) | 145.3 (129.3 to 162.2) | 4.8 (2.7 to 8) |  | 0.0081 ( 0.0063 to 0.0100) | 0.0081 ( 0.0062 to 0.0100) | 0.0115 (-0.0014 to 0.0262) |
| Kazakhstan | 303.8 (267.5 to 341.4) | 163 (145 to 181.8) | 5.5 (3.1 to 8.5) |  | 0.0046 ( 0.0037 to 0.0059) | 0.0047 ( 0.0038 to 0.0060) | 0.0081 (-0.0079 to 0.0240) |
| Kenya | 295.2 (260.7 to 330.9) | 158.9 (141.5 to 177.3) | 5.3 (3.4 to 7.7) |  | -0.0134 (-0.0137 to -0.0132) | -0.0132 (-0.0135 to -0.0130) | -0.0106 (-0.0148 to -0.0079) |
| Kiribati | 355.7 (315.5 to 401.6) | 190.6 (170.4 to 213.2) | 6.3 (3.6 to 10) |  | 0.0083 ( 0.0075 to 0.0090) | 0.0082 ( 0.0075 to 0.0090) | 0.0046 (-0.0252 to 0.0297) |
| Kuwait | 272.6 (239.9 to 305) | 146.7 (130.6 to 163.8) | 4.9 (2.7 to 8) |  | -0.0036 (-0.0072 to 0.0004) | -0.0034 (-0.0070 to 0.0006) | -0.0065 (-0.0380 to 0.0161) |
| Kyrgyzstan | 303.6 (267.3 to 341.1) | 162.8 (144.8 to 181.6) | 5.5 (3.1 to 8.7) |  | -0.0079 (-0.0084 to -0.0071) | -0.0079 (-0.0084 to -0.0071) | -0.0144 (-0.0411 to 0.0037) |
| Lao People's Democratic Republic | 379.2 (335.3 to 427) | 203.4 (180.8 to 227.7) | 6.8 (4 to 10.7) |  | -0.0304 (-0.0308 to -0.0300) | -0.0305 (-0.0309 to -0.0301) | -0.0154 (-0.0415 to 0.0146) |
| Latvia | 302.4 (266.3 to 339.8) | 162.2 (144.3 to 180.9) | 5.4 (3 to 8.6) |  | -0.0038 (-0.0047 to -0.0028) | -0.0038 (-0.0047 to -0.0027) | 0.0074 (-0.0103 to 0.0254) |
| Lebanon | 270 (237.5 to 301.9) | 145.3 (129.3 to 162.2) | 4.8 (2.7 to 7.6) |  | -0.0464 (-0.0491 to -0.0440) | -0.0464 (-0.0491 to -0.0440) | -0.0488 (-0.0834 to -0.0161) |
| Lesotho | 292.7 (258.7 to 329.7) | 157.2 (140.4 to 175.3) | 5.2 (2.8 to 8.4) |  | -0.0800 (-0.0806 to -0.0793) | -0.0801 (-0.0807 to -0.0793) | -0.0554 (-0.0737 to -0.0370) |
| Liberia | 292.6 (258.7 to 329.6) | 157.2 (140.4 to 175.2) | 5.2 (2.8 to 8.1) |  | -0.0419 (-0.0600 to -0.0203) | -0.0420 (-0.0600 to -0.0205) | -0.0208 (-0.0481 to 0.0200) |
| Libya | 273.3 (240.5 to 305.8) | 147.1 (130.9 to 164.2) | 4.9 (2.7 to 8) |  | -0.0003 (-0.0020 to 0.0013) | 0.0000 (-0.0017 to 0.0016) | 0.0062 (-0.0223 to 0.0294) |
| Lithuania | 302.5 (266.4 to 339.9) | 162.3 (144.3 to 180.9) | 5.4 (3 to 8.6) |  | -0.0060 (-0.0066 to -0.0053) | -0.0060 (-0.0066 to -0.0053) | -0.0209 (-0.0404 to -0.0012) |
| Luxembourg | 368.8 (326.2 to 412.2) | 196.8 (175.6 to 219.1) | 6.5 (3.7 to 10.2) |  | 0.0022 ( 0.0011 to 0.0031) | 0.0016 ( 0.0008 to 0.0024) | 0.0144 (-0.0022 to 0.0314) |
| Madagascar | 295.1 (260.8 to 330.4) | 158.6 (141 to 177.2) | 5.3 (2.9 to 8.3) |  | 0.0025 ( 0.0023 to 0.0026) | 0.0024 ( 0.0023 to 0.0026) | 0.0267 ( 0.0061 to 0.0475) |
| Malawi | 295.8 (261.4 to 331.2) | 159 (141.3 to 177.6) | 5.3 (2.9 to 8.7) |  | 0.0005 (-0.0012 to 0.0023) | 0.0005 (-0.0012 to 0.0023) | 0.0266 ( 0.0062 to 0.0454) |
| Malaysia | 377.6 (333.7 to 425.1) | 202.5 (180 to 226.6) | 6.8 (3.9 to 11) |  | -0.0164 (-0.0166 to -0.0161) | -0.0162 (-0.0164 to -0.0160) | -0.0244 (-0.0402 to -0.0089) |
| Maldives | 371 (327.8 to 417.6) | 198.9 (176.6 to 222.6) | 6.6 (3.7 to 10.6) |  | -0.0831 (-0.0852 to -0.0809) | -0.0834 (-0.0855 to -0.0812) | -0.0725 (-0.0984 to -0.0446) |
| Mali | 293.4 (259.3 to 330.5) | 157.6 (140.8 to 175.7) | 5.2 (2.9 to 8.3) |  | -0.0251 (-0.0260 to -0.0242) | -0.0252 (-0.0261 to -0.0243) | -0.0320 (-0.0679 to 0.0085) |
| Malta | 368.7 (326.2 to 412.1) | 196.7 (175.6 to 219.1) | 6.6 (3.7 to 10.5) |  | 0.0001 (-0.0006 to 0.0006) | 0.0002 (-0.0004 to 0.0006) | 0.0002 (-0.0179 to 0.0183) |
| Marshall Islands | 353.5 (313.6 to 399.2) | 189.5 (169.4 to 211.9) | 6.3 (3.6 to 9.9) |  | -0.0026 (-0.0032 to -0.0022) | -0.0027 (-0.0033 to -0.0023) | -0.0137 (-0.0261 to -0.0010) |
| Mauritania | 295 (260.6 to 332.4) | 158.5 (141.6 to 176.7) | 5.3 (2.9 to 8.5) |  | 0.0090 ( 0.0085 to 0.0095) | 0.0090 ( 0.0085 to 0.0095) | -0.0001 (-0.0175 to 0.0175) |
| Mauritius | 379.2 (335.2 to 427) | 203.3 (180.7 to 227.6) | 6.8 (3.9 to 10.6) |  | 0.0050 ( 0.0047 to 0.0053) | 0.0050 ( 0.0048 to 0.0053) | 0.0052 (-0.0106 to 0.0212) |
| Mexico | 306.3 (270.3 to 345.2) | 164.6 (146.4 to 184.1) | 5.5 (3.5 to 8.1) |  | -0.0227 (-0.0230 to -0.0225) | -0.0228 (-0.0231 to -0.0225) | -0.0219 (-0.0262 to -0.0167) |
| Micronesia (Federated States of) | 352.2 (312.4 to 397.7) | 188.8 (168.8 to 211.1) | 6.3 (3.5 to 9.9) |  | -0.0230 (-0.0233 to -0.0225) | -0.0232 (-0.0236 to -0.0228) | -0.0141 (-0.0300 to 0.0024) |
| Monaco | 368.8 (326.3 to 412.2) | 196.8 (175.6 to 219.1) | 6.5 (3.7 to 10) |  | 0.0040 ( 0.0028 to 0.0052) | 0.0034 ( 0.0024 to 0.0043) | 0.0016 (-0.0254 to 0.0324) |
| Mongolia | 303.8 (267.5 to 341.4) | 163 (144.9 to 181.8) | 5.4 (3 to 8.8) |  | -0.0080 (-0.0085 to -0.0074) | -0.0080 (-0.0085 to -0.0074) | -0.0121 (-0.0319 to 0.0073) |
| Montenegro | 301.3 (265.4 to 338.6) | 161.6 (143.8 to 180.2) | 5.4 (3 to 8.9) |  | -0.0027 (-0.0032 to -0.0022) | -0.0026 (-0.0032 to -0.0021) | -0.0338 (-0.0530 to -0.0145) |
| Morocco | 273.5 (240.6 to 305.9) | 147.1 (131 to 164.3) | 4.9 (2.7 to 7.9) |  | -0.0220 (-0.0223 to -0.0217) | -0.0218 (-0.0222 to -0.0215) | -0.0157 (-0.0364 to 0.0053) |
| Mozambique | 298.3 (263.5 to 334) | 160.3 (142.5 to 179.1) | 5.3 (2.9 to 8.5) |  | -0.0235 (-0.0248 to -0.0222) | -0.0235 (-0.0248 to -0.0222) | -0.0083 (-0.0355 to 0.0215) |
| Myanmar | 380.2 (336.1 to 428.2) | 203.9 (181.2 to 228.3) | 6.8 (3.9 to 10.7) |  | -0.0070 (-0.0071 to -0.0068) | -0.0070 (-0.0071 to -0.0069) | -0.0087 (-0.0252 to 0.0080) |
| Namibia | 293.2 (259.2 to 330.3) | 157.5 (140.7 to 175.6) | 5.2 (3 to 8.4) |  | -0.0100 (-0.0103 to -0.0098) | -0.0101 (-0.0103 to -0.0098) | 0.0010 (-0.0229 to 0.0252) |
| Nauru | 352.4 (312.7 to 398) | 188.9 (168.9 to 211.3) | 6.4 (3.6 to 10.3) |  | -0.0170 (-0.0174 to -0.0164) | -0.0169 (-0.0174 to -0.0164) | 0.0076 (-0.0120 to 0.0276) |
| Nepal | 273.6 (243.8 to 308) | 146.9 (131.3 to 164.3) | 4.9 (2.8 to 8) |  | 0.0019 ( 0.0011 to 0.0027) | 0.0019 ( 0.0011 to 0.0027) | 0.0266 ( 0.0002 to 0.0491) |
| Netherlands | 368.4 (324.9 to 413.2) | 196.7 (175.6 to 219.1) | 6.6 (3.8 to 10.4) |  | -0.0035 (-0.0037 to -0.0033) | -0.0016 (-0.0017 to -0.0015) | -0.0066 (-0.0213 to 0.0085) |
| New Zealand | 384.4 (340.2 to 430.3) | 205.7 (183.1 to 230.4) | 6.9 (4 to 10.6) |  | -0.0055 (-0.0061 to -0.0050) | -0.0065 (-0.0073 to -0.0058) | -0.0043 (-0.0162 to 0.0081) |
| Nicaragua | 303.3 (267 to 340.8) | 162.7 (144.7 to 181.4) | 5.4 (3.1 to 9) |  | -0.0509 (-0.0512 to -0.0507) | -0.0511 (-0.0514 to -0.0509) | -0.0312 (-0.0484 to -0.0140) |
| Niger | 294.3 (260 to 331.6) | 158.1 (141.2 to 176.3) | 5.3 (2.9 to 8.5) |  | -0.0280 (-0.0287 to -0.0273) | -0.0278 (-0.0285 to -0.0272) | 0.0109 (-0.0158 to 0.0393) |
| Nigeria | 298.3 (263.8 to 336.3) | 160.5 (143.4 to 179.4) | 5.3 (3.4 to 7.7) |  | -0.0040 (-0.0054 to -0.0026) | -0.0039 (-0.0053 to -0.0024) | 0.0097 ( 0.0046 to 0.0146) |
| Niue | 348.9 (309.5 to 394) | 187 (167.4 to 209.2) | 6.3 (3.6 to 9.9) |  | 0.0148 ( 0.0119 to 0.0175) | 0.0151 ( 0.0121 to 0.0178) | -0.0030 (-0.0251 to 0.0175) |
| North Macedonia | 301.7 (265.6 to 339) | 161.8 (143.9 to 180.4) | 5.4 (3.1 to 8.6) |  | -0.0139 (-0.0145 to -0.0133) | -0.0140 (-0.0145 to -0.0134) | -0.0283 (-0.0429 to -0.0136) |
| Northern Mariana Islands | 351.5 (311.8 to 396.9) | 188.4 (168.5 to 210.7) | 6.3 (3.5 to 10) |  | -0.1030 (-0.1274 to -0.0721) | -0.1034 (-0.1275 to -0.0729) | -0.1243 (-0.1547 to -0.0852) |
| Norway | 370.7 (327.2 to 416.9) | 198.2 (177.1 to 222.4) | 6.6 (4.1 to 9.8) |  | 0.0009 ( 0.0007 to 0.0011) | 0.0006 ( 0.0005 to 0.0008) | 0.0017 (-0.0047 to 0.0082) |
| Oman | 267.8 (235.7 to 299.6) | 144.1 (128.3 to 160.8) | 4.8 (2.6 to 7.9) |  | 0.0540 ( 0.0513 to 0.0567) | 0.0540 ( 0.0513 to 0.0566) | 0.0644 ( 0.0380 to 0.0955) |
| Pakistan | 269.3 (238 to 301.1) | 144.8 (129 to 161.4) | 4.8 (2.9 to 7.3) |  | 0.0152 ( 0.0147 to 0.0157) | 0.0153 ( 0.0148 to 0.0158) | 0.0177 ( 0.0026 to 0.0328) |
| Palau | 346.3 (307.4 to 391) | 185.6 (165.9 to 207.4) | 6.2 (3.5 to 9.5) |  | -0.0387 (-0.0448 to -0.0323) | -0.0410 (-0.0505 to -0.0322) | -0.0365 (-0.0657 to -0.0075) |
| Palestine | 272.7 (239.9 to 305.1) | 146.7 (130.6 to 163.8) | 4.9 (2.7 to 7.8) |  | -0.0017 (-0.0026 to -0.0009) | -0.0018 (-0.0026 to -0.0009) | 0.0050 (-0.0113 to 0.0217) |
| Panama | 302.8 (266.6 to 340.2) | 162.4 (144.5 to 181.1) | 5.5 (3.1 to 9) |  | -0.0164 (-0.0171 to -0.0156) | -0.0164 (-0.0171 to -0.0157) | 0.0024 (-0.0176 to 0.0224) |
| Papua New Guinea | 350.2 (310.7 to 395.5) | 187.7 (167.9 to 209.9) | 6.2 (3.5 to 9.9) |  | -0.0145 (-0.0151 to -0.0140) | -0.0145 (-0.0151 to -0.0138) | -0.0010 (-0.0204 to 0.0189) |
| Paraguay | 303.1 (266.9 to 340.6) | 162.6 (144.6 to 181.3) | 5.4 (3 to 9) |  | -0.0214 (-0.0216 to -0.0212) | -0.0215 (-0.0217 to -0.0213) | 0.0394 ( 0.0066 to 0.0791) |
| Peru | 300.6 (264.8 to 337.8) | 161.3 (143.4 to 179.8) | 5.4 (3 to 8.7) |  | -0.0568 (-0.0576 to -0.0560) | -0.0569 (-0.0577 to -0.0561) | -0.0552 (-0.1109 to 0.0123) |
| Philippines | 379.9 (334.5 to 426) | 204 (182.8 to 227.6) | 6.8 (4.3 to 10.1) |  | -0.0137 (-0.0139 to -0.0135) | -0.0137 (-0.0139 to -0.0135) | -0.0015 (-0.0058 to 0.0036) |
| Poland | 304.2 (268.5 to 342.8) | 163.5 (145.5 to 182.9) | 5.5 (3.4 to 8.2) |  | -0.0031 (-0.0033 to -0.0030) | -0.0031 (-0.0033 to -0.0030) | -0.0025 (-0.0128 to 0.0047) |
| Portugal | 368.7 (326.1 to 412) | 196.7 (175.5 to 219) | 6.6 (3.8 to 10.4) |  | 0.0002 ( 0.0001 to 0.0004) | 0.0001 (-0.0001 to 0.0002) | 0.0271 (-0.0038 to 0.0592) |
| Puerto Rico | 304 (267.6 to 341.6) | 163.1 (145 to 181.9) | 5.5 (3 to 8.8) |  | -0.0106 (-0.0126 to -0.0092) | -0.0106 (-0.0127 to -0.0092) | -0.0013 (-0.0188 to 0.0168) |
| Qatar | 254.4 (224.2 to 285.1) | 136.9 (121.8 to 152.6) | 4.5 (2.5 to 7.3) |  | -0.0084 (-0.0169 to 0.0020) | -0.0088 (-0.0174 to 0.0014) | -0.0128 (-0.0385 to 0.0113) |
| Republic of Korea | 379.6 (335.1 to 425) | 202.8 (180.7 to 226) | 6.8 (3.8 to 10.8) |  | -0.0012 (-0.0013 to -0.0011) | -0.0012 (-0.0014 to -0.0010) | -0.0088 (-0.0239 to 0.0066) |
| Republic of Moldova | 302.5 (266.3 to 339.9) | 162.2 (144.3 to 180.9) | 5.5 (2.9 to 8.9) |  | -0.0259 (-0.0262 to -0.0255) | -0.0260 (-0.0264 to -0.0257) | -0.0082 (-0.0279 to 0.0116) |
| Romania | 302.3 (266.2 to 339.7) | 162.2 (144.2 to 180.8) | 5.4 (3.1 to 8.6) |  | -0.0138 (-0.0143 to -0.0134) | -0.0139 (-0.0143 to -0.0135) | 0.0020 (-0.0142 to 0.0183) |
| Russian Federation | 304.3 (268.6 to 343) | 163.5 (145.5 to 182.9) | 5.5 (3.5 to 8) |  | -0.0045 (-0.0049 to -0.0041) | -0.0045 (-0.0048 to -0.0043) | 0.0002 (-0.0026 to 0.0031) |
| Rwanda | 294.5 (260.2 to 329.7) | 158.3 (140.7 to 176.8) | 5.3 (3 to 8.5) |  | -0.0146 (-0.0341 to 0.0170) | -0.0146 (-0.0341 to 0.0169) | 0.0186 (-0.0153 to 0.0604) |
| Saint Kitts and Nevis | 305.2 (268.6 to 342.9) | 163.7 (145.6 to 182.7) | 5.5 (3.1 to 8.6) |  | 0.0070 ( 0.0067 to 0.0073) | 0.0070 ( 0.0067 to 0.0073) | 0.0237 ( 0.0068 to 0.0411) |
| Saint Lucia | 302.6 (266.5 to 340) | 162.3 (144.4 to 181) | 5.4 (3.1 to 8.7) |  | -0.0361 (-0.0368 to -0.0353) | -0.0362 (-0.0369 to -0.0355) | -0.0346 (-0.0509 to -0.0185) |
| Saint Vincent and the Grenadines | 302.2 (266.1 to 339.6) | 162.1 (144.2 to 180.7) | 5.4 (3.1 to 8.7) |  | -0.0157 (-0.0162 to -0.0153) | -0.0158 (-0.0163 to -0.0153) | -0.0191 (-0.0379 to 0.0002) |
| Samoa | 352 (312.2 to 397.5) | 188.6 (168.7 to 211) | 6.3 (3.5 to 10.1) |  | 0.0482 ( 0.0476 to 0.0488) | 0.0483 ( 0.0478 to 0.0490) | 0.0578 ( 0.0301 to 0.0850) |
| San Marino | 368.5 (326 to 411.9) | 196.7 (175.5 to 219) | 6.5 (3.7 to 10.3) |  | 0.0006 ( 0.0004 to 0.0008) | 0.0000 (-0.0001 to 0.0001) | -0.0004 (-0.0239 to 0.0193) |
| Sao Tome and Principe | 292.7 (258.8 to 329.7) | 157.2 (140.4 to 175.3) | 5.3 (2.9 to 8.3) |  | 0.0080 ( 0.0076 to 0.0084) | 0.0079 ( 0.0075 to 0.0083) | 0.0224 ( 0.0078 to 0.0370) |
| Saudi Arabia | 265.4 (233.6 to 296.8) | 142.8 (127.2 to 159.3) | 4.7 (2.5 to 7.9) |  | -0.0189 (-0.0201 to -0.0175) | -0.0186 (-0.0198 to -0.0172) | -0.0354 (-0.0680 to -0.0044) |
| Senegal | 289.7 (256.2 to 326) | 155.6 (138.9 to 173.5) | 5.2 (2.9 to 8.3) |  | -0.0742 (-0.0751 to -0.0734) | -0.0741 (-0.0750 to -0.0733) | -0.0306 (-0.0513 to -0.0094) |
| Serbia | 300.4 (264.5 to 337.5) | 161.1 (143.3 to 179.6) | 5.4 (3 to 8.5) |  | -0.0294 (-0.0319 to -0.0267) | -0.0293 (-0.0318 to -0.0267) | -0.0526 (-0.0819 to -0.0211) |
| Seychelles | 377 (333.1 to 424.3) | 202.1 (179.6 to 226.2) | 6.8 (3.9 to 10.7) |  | -0.0257 (-0.0263 to -0.0250) | -0.0258 (-0.0264 to -0.0252) | -0.0458 (-0.0626 to -0.0286) |
| Sierra Leone | 294.6 (260.3 to 332) | 158.3 (141.4 to 176.5) | 5.3 (2.9 to 8.4) |  | -0.0235 (-0.0263 to -0.0196) | -0.0236 (-0.0265 to -0.0198) | -0.0031 (-0.0217 to 0.0225) |
| Singapore | 379.7 (335.1 to 425.3) | 202.9 (180.7 to 226.1) | 6.8 (3.9 to 10.8) |  | 0.0108 ( 0.0090 to 0.0126) | 0.0099 ( 0.0089 to 0.0110) | 0.0256 ( 0.0046 to 0.0466) |
| Slovakia | 302.6 (266.4 to 340) | 162.3 (144.4 to 181) | 5.4 (3.1 to 8.5) |  | -0.0079 (-0.0081 to -0.0077) | -0.0077 (-0.0079 to -0.0075) | 0.0025 (-0.0130 to 0.0178) |
| Slovenia | 302.1 (266 to 339.4) | 162 (144.1 to 180.6) | 5.4 (3 to 8.8) |  | -0.0180 (-0.0188 to -0.0173) | -0.0179 (-0.0186 to -0.0172) | -0.0112 (-0.0282 to 0.0061) |
| Solomon Islands | 352.3 (312.6 to 397.9) | 188.8 (168.9 to 211.2) | 6.3 (3.5 to 9.8) |  | -0.0099 (-0.0103 to -0.0094) | -0.0099 (-0.0104 to -0.0094) | -0.0010 (-0.0196 to 0.0175) |
| Somalia | 289.9 (256.3 to 324.5) | 155.8 (138.6 to 173.9) | 5.2 (2.8 to 8.3) |  | 0.0261 ( 0.0248 to 0.0274) | 0.0259 ( 0.0246 to 0.0273) | 0.0076 (-0.0242 to 0.0281) |
| South Africa | 291.9 (258.1 to 329.1) | 157.1 (140.3 to 175.4) | 5.2 (3.2 to 7.6) |  | -0.0404 (-0.0409 to -0.0400) | -0.0404 (-0.0409 to -0.0400) | -0.0416 (-0.0486 to -0.0347) |
| South Sudan | 293.6 (259.5 to 328.7) | 157.8 (140.3 to 176.2) | 5.2 (2.9 to 8.5) |  | 0.0430 ( 0.0386 to 0.0473) | 0.0434 ( 0.0390 to 0.0477) | 0.0444 ( 0.0178 to 0.0733) |
| Spain | 368.7 (326.2 to 412.1) | 196.8 (175.6 to 219.1) | 6.6 (3.7 to 10.4) |  | 0.0005 ( 0.0004 to 0.0006) | 0.0005 ( 0.0004 to 0.0006) | -0.0354 (-0.0535 to -0.0171) |
| Sri Lanka | 379.8 (335.7 to 427.7) | 203.7 (181 to 228) | 6.8 (3.9 to 10.5) |  | -0.0437 (-0.0474 to -0.0408) | -0.0441 (-0.0478 to -0.0413) | -0.0655 (-0.0923 to -0.0425) |
| Sudan | 272.4 (239.6 to 304.6) | 146.5 (130.4 to 163.6) | 4.9 (2.6 to 8) |  | -0.0395 (-0.0408 to -0.0380) | -0.0395 (-0.0408 to -0.0380) | -0.0242 (-0.0446 to -0.0040) |
| Suriname | 302.9 (266.7 to 340.4) | 162.5 (144.5 to 181.2) | 5.4 (3 to 8.7) |  | 0.0129 ( 0.0122 to 0.0136) | 0.0131 ( 0.0123 to 0.0138) | 0.0131 (-0.0026 to 0.0295) |
| Sweden | 370.8 (327.3 to 417) | 198.2 (177.1 to 222.4) | 6.6 (3.9 to 10.2) |  | 0.0021 ( 0.0019 to 0.0022) | 0.0015 ( 0.0013 to 0.0016) | 0.0002 (-0.0196 to 0.0180) |
| Switzerland | 369 (326.4 to 412.4) | 196.8 (175.7 to 219.2) | 6.6 (3.7 to 10.6) |  | 0.0009 ( 0.0008 to 0.0010) | 0.0007 ( 0.0006 to 0.0008) | 0.0371 ( 0.0193 to 0.0550) |
| Syrian Arab Republic | 280.8 (246.8 to 314) | 151.1 (134.4 to 168.7) | 5 (2.7 to 8) |  | 0.0842 ( 0.0764 to 0.0906) | 0.0843 ( 0.0765 to 0.0908) | 0.0736 ( 0.0347 to 0.1005) |
| Taiwan (Province of China) | 333.6 (294.9 to 373.6) | 178.8 (159.6 to 199.9) | 6 (3.3 to 9.5) |  | -0.0131 (-0.0152 to -0.0119) | -0.0103 (-0.0137 to -0.0085) | -0.0396 (-0.0539 to -0.0251) |
| Tajikistan | 302.6 (266.4 to 340) | 162.3 (144.4 to 181) | 5.4 (3 to 8.7) |  | -0.0307 (-0.0319 to -0.0289) | -0.0308 (-0.0320 to -0.0290) | -0.0615 (-0.0871 to -0.0326) |
| Thailand | 379.3 (335.3 to 427.1) | 203.4 (180.8 to 227.7) | 6.8 (3.9 to 10.7) |  | 0.0053 ( 0.0033 to 0.0070) | 0.0053 ( 0.0034 to 0.0071) | 0.0171 ( 0.0038 to 0.0309) |
| Timor-Leste | 378.8 (334.8 to 426.5) | 203.2 (180.6 to 227.4) | 6.8 (3.9 to 10.6) |  | 0.0200 ( 0.0188 to 0.0212) | 0.0199 ( 0.0187 to 0.0211) | 0.0488 ( 0.0223 to 0.0783) |
| Togo | 290.7 (257.1 to 327.3) | 156.2 (139.4 to 174.1) | 5.2 (2.9 to 8.3) |  | -0.0368 (-0.0384 to -0.0355) | -0.0369 (-0.0384 to -0.0355) | -0.0163 (-0.0464 to 0.0082) |
| Tokelau | 349.6 (310.2 to 394.9) | 187.4 (167.7 to 209.6) | 6.3 (3.5 to 9.9) |  | -0.0177 (-0.0358 to -0.0057) | -0.0175 (-0.0352 to -0.0056) | -0.0036 (-0.0296 to 0.0157) |
| Tonga | 353 (313.2 to 398.6) | 189.2 (169.2 to 211.6) | 6.3 (3.6 to 10) |  | 0.0111 ( 0.0107 to 0.0115) | 0.0111 ( 0.0107 to 0.0114) | 0.0088 (-0.0072 to 0.0251) |
| Trinidad and Tobago | 303.4 (267.2 to 341) | 162.8 (144.8 to 181.5) | 5.4 (3.1 to 8.7) |  | -0.0086 (-0.0089 to -0.0084) | -0.0087 (-0.0089 to -0.0084) | -0.0223 (-0.0417 to -0.0048) |
| Tunisia | 273.3 (240.4 to 305.7) | 147.1 (130.9 to 164.2) | 4.9 (2.8 to 7.9) |  | -0.0055 (-0.0058 to -0.0052) | -0.0053 (-0.0057 to -0.0050) | 0.0044 (-0.0149 to 0.0239) |
| Turkey | 263.6 (232.3 to 296.3) | 141.7 (125.7 to 158.7) | 4.8 (2.6 to 7.7) |  | -0.0051 (-0.0059 to -0.0044) | -0.0048 (-0.0056 to -0.0040) | 0.0127 (-0.0029 to 0.0282) |
| Turkmenistan | 300.4 (264.5 to 337.5) | 161.1 (143.3 to 179.6) | 5.4 (3 to 8.6) |  | -0.0415 (-0.0419 to -0.0411) | -0.0416 (-0.0420 to -0.0413) | -0.0300 (-0.0546 to -0.0105) |
| Tuvalu | 349 (309.7 to 394.2) | 187.1 (167.3 to 209.2) | 6.3 (3.5 to 9.8) |  | -0.0607 (-0.0613 to -0.0600) | -0.0610 (-0.0616 to -0.0603) | -0.0436 (-0.0686 to -0.0220) |
| Uganda | 294.2 (260 to 329.3) | 158.1 (140.5 to 176.6) | 5.2 (2.9 to 8.2) |  | -0.0300 (-0.0304 to -0.0297) | -0.0299 (-0.0303 to -0.0295) | -0.0019 (-0.0448 to 0.0434) |
| Ukraine | 303.9 (268.2 to 342.4) | 163.3 (145.3 to 182.7) | 5.4 (3.2 to 8.9) |  | -0.0159 (-0.0162 to -0.0157) | -0.0160 (-0.0162 to -0.0158) | -0.0239 (-0.0398 to -0.0077) |
| United Arab Emirates | 271.6 (238.9 to 303.8) | 146.1 (130.1 to 163.1) | 4.9 (2.7 to 8) |  | 0.1138 ( 0.0945 to 0.1295) | 0.1141 ( 0.0949 to 0.1294) | 0.1024 ( 0.0509 to 0.1351) |
| United Kingdom | 370 (328.8 to 414.2) | 197.8 (176.6 to 220.8) | 6.6 (4.2 to 9.6) |  | 0.0002 ( 0.0001 to 0.0003) | 0.0003 ( 0.0002 to 0.0003) | -0.0032 (-0.0072 to 0.0031) |
| United Republic of Tanzania | 298.2 (262.8 to 334.8) | 160.4 (143 to 178.6) | 5.3 (3 to 8.6) |  | -0.0184 (-0.0191 to -0.0178) | -0.0163 (-0.0167 to -0.0158) | 0.0386 ( 0.0199 to 0.0573) |
| United States of America | 436.1 (393.6 to 480.7) | 233.9 (212.2 to 256.4) | 7.7 (5 to 11.4) |  | -0.4081 (-0.4454 to -0.3650) | -0.3747 (-0.4090 to -0.3353) | -0.3930 (-0.4245 to -0.3574) |
| United States Virgin Islands | 304.9 (268.4 to 342.6) | 163.5 (145.5 to 182.5) | 5.4 (3.1 to 8.5) |  | -0.0109 (-0.0119 to -0.0099) | -0.0110 (-0.0119 to -0.0100) | -0.0438 (-0.0671 to -0.0216) |
| Uruguay | 383.3 (338.7 to 429.3) | 204.7 (182.2 to 227.8) | 6.8 (3.9 to 10.6) |  | -0.0018 (-0.0018 to -0.0017) | -0.0018 (-0.0020 to -0.0017) | -0.0122 (-0.0289 to 0.0014) |
| Uzbekistan | 302.9 (266.7 to 340.3) | 162.5 (144.5 to 181.2) | 5.4 (3 to 8.7) |  | -0.0229 (-0.0230 to -0.0227) | -0.0229 (-0.0230 to -0.0228) | -0.0200 (-0.0359 to -0.0035) |
| Vanuatu | 353.9 (314 to 399.6) | 189.7 (169.6 to 212.2) | 6.4 (3.6 to 10.1) |  | -0.0056 (-0.0064 to -0.0049) | -0.0056 (-0.0064 to -0.0049) | 0.0051 (-0.0146 to 0.0249) |
| Venezuela (Bolivarian Republic of) | 307.7 (270.8 to 345.6) | 165 (146.8 to 184.3) | 5.5 (3.1 to 8.9) |  | 0.0332 ( 0.0291 to 0.0375) | 0.0334 ( 0.0293 to 0.0377) | 0.0430 ( 0.0133 to 0.0695) |
| Viet Nam | 377.5 (333.6 to 425) | 202.5 (179.9 to 226.6) | 6.8 (3.8 to 10.8) |  | -0.0296 (-0.0299 to -0.0294) | -0.0297 (-0.0299 to -0.0295) | -0.0015 (-0.0250 to 0.0259) |
| Yemen | 273.8 (240.9 to 306.3) | 147.3 (131.1 to 164.5) | 4.9 (2.7 to 7.8) |  | 0.0070 ( 0.0060 to 0.0079) | 0.0067 ( 0.0058 to 0.0076) | -0.0093 (-0.0250 to 0.0164) |
| Zambia | 295.8 (261.4 to 331.2) | 159 (141.3 to 177.6) | 5.3 (2.9 to 8.5) |  | -0.0130 (-0.0132 to -0.0128) | -0.0130 (-0.0132 to -0.0128) | -0.0026 (-0.0215 to 0.0172) |
| Zimbabwe | 294.6 (260.3 to 332) | 158.3 (141.4 to 176.5) | 5.3 (2.9 to 8.6) |  | -0.0028 (-0.0032 to -0.0024) | -0.0028 (-0.0032 to -0.0024) | 0.0045 (-0.0177 to 0.0270) |

AAPC, Average annual percentage change; DALYs, disability adjusted life-years.


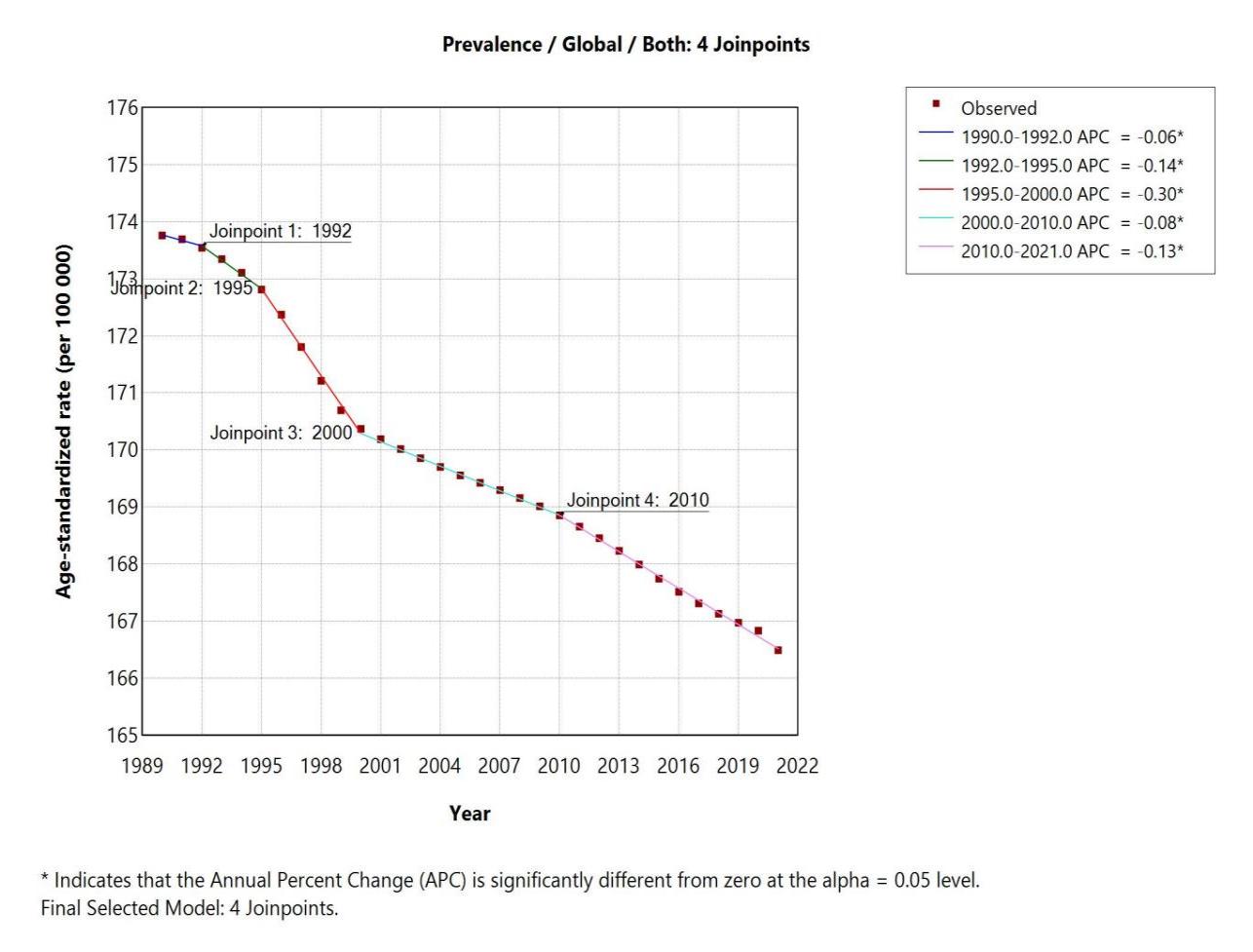


**Figure S1. Joinpoint regression analysis of global ASPR of alopecia areata adolescents and young adults aged 10-24 years from 1990 to 2021.** ASPR, Age-standardized prevalence rate.


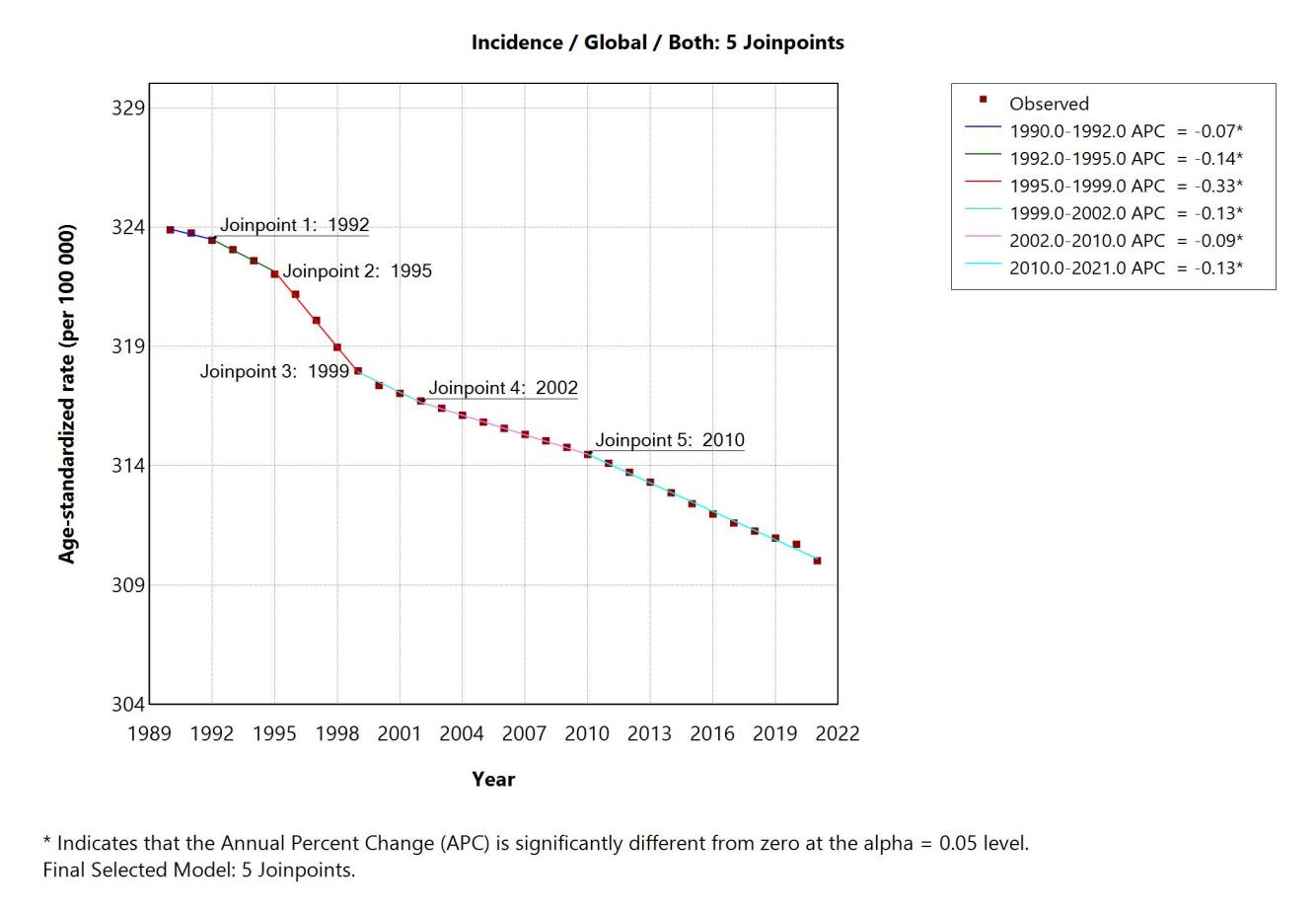


**Figure S2. Joinpoint regression analysis of global ASIR of alopecia areata in adolescents and young adults aged 10-24 years from 1990 to 2021.** ASIR, Age-standardized incidence rate.


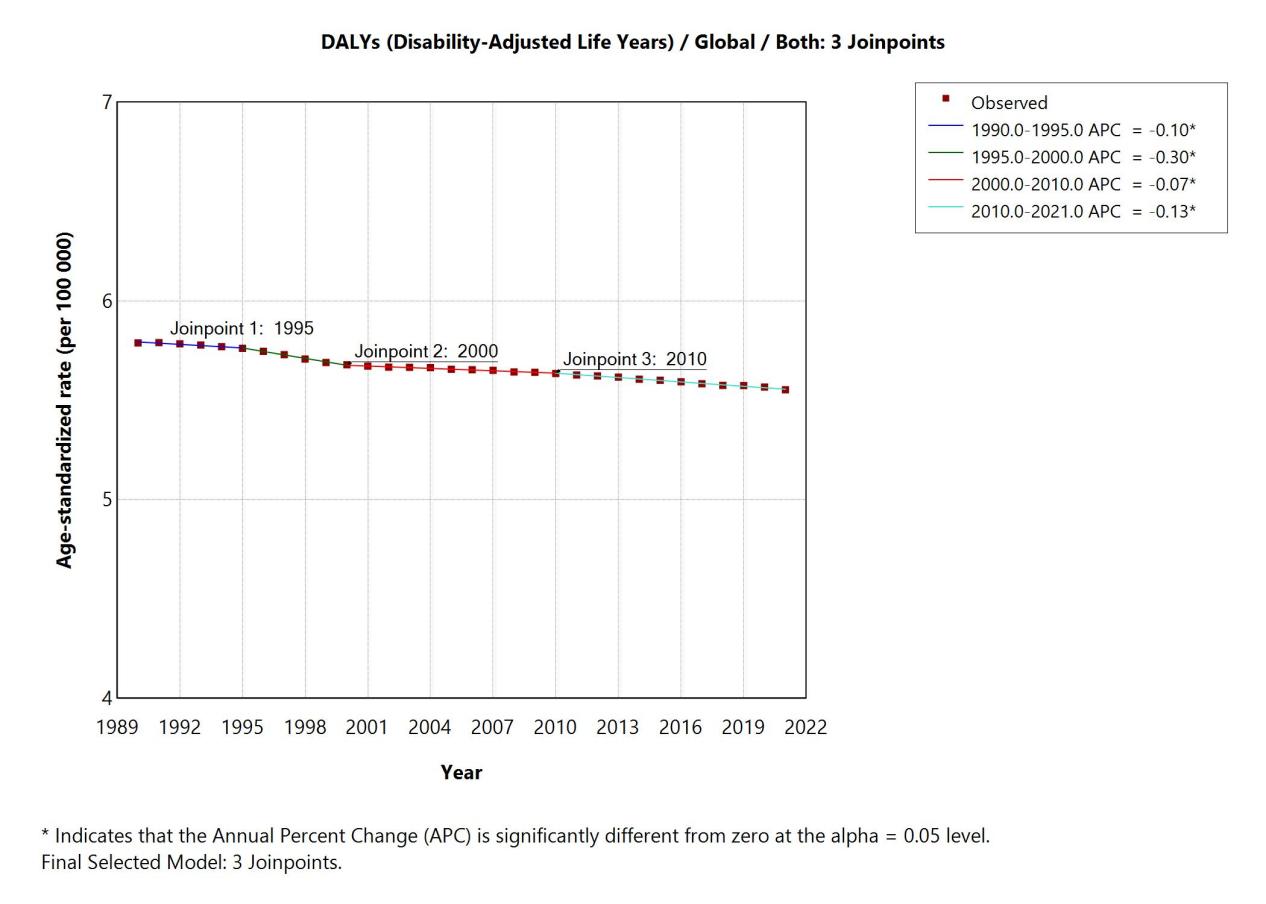


**Figure S3. Joinpoint regression analysis of global ASDR of alopecia areata in adolescents and young adults aged 10-24 years from 1990 to 2021.** ASDR, Age-standardized DALYs rate; DALYs, disability adjusted life-years.


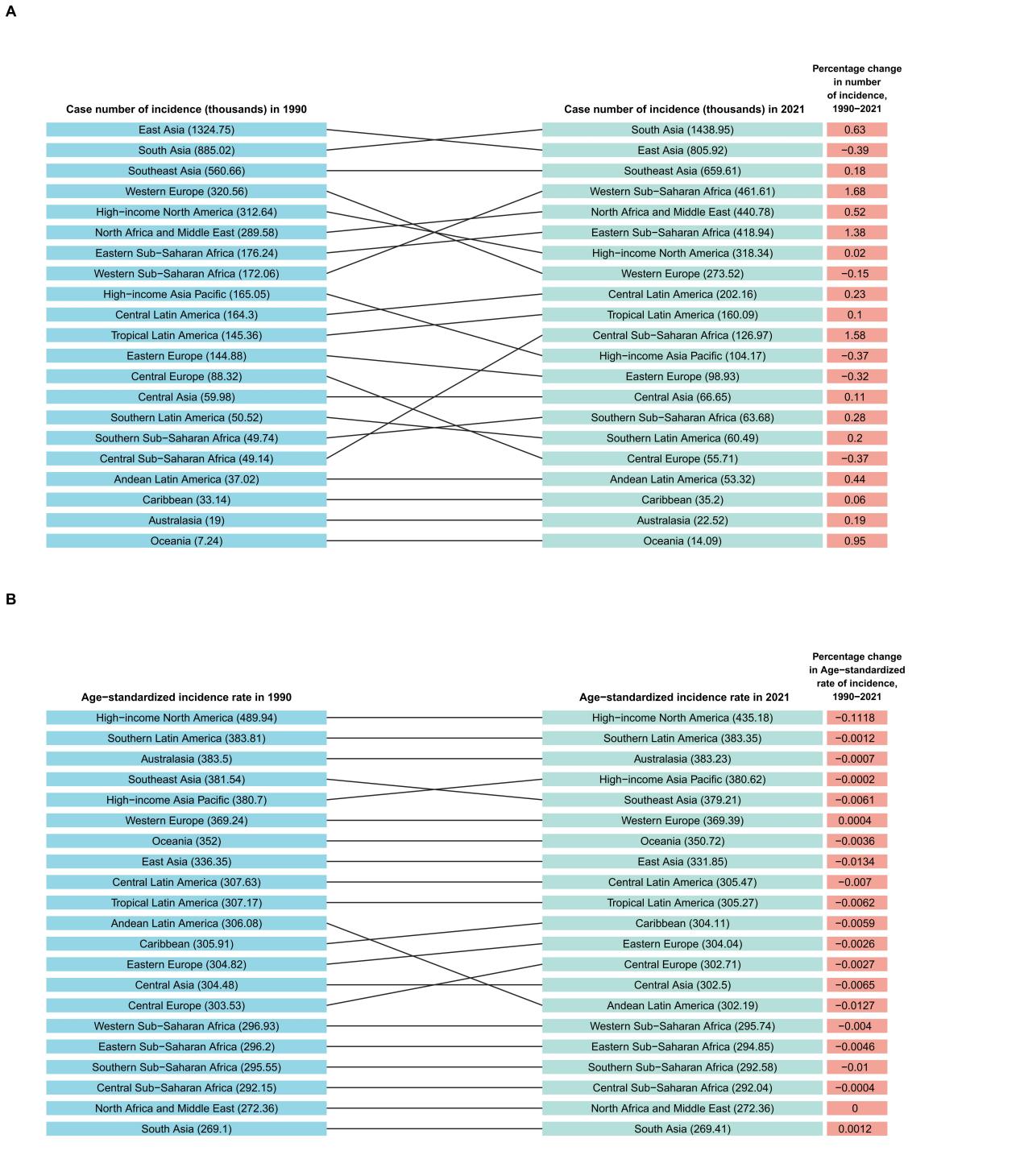


**Figure S4. The number of incident cases (A) and ASIR (b) for alopecia areata among adolescents and young adults across 21 GBD regions from 1990 to 2021.** GBD, Global Burden of Disease; ASIR, Age-standardized incidence rate.


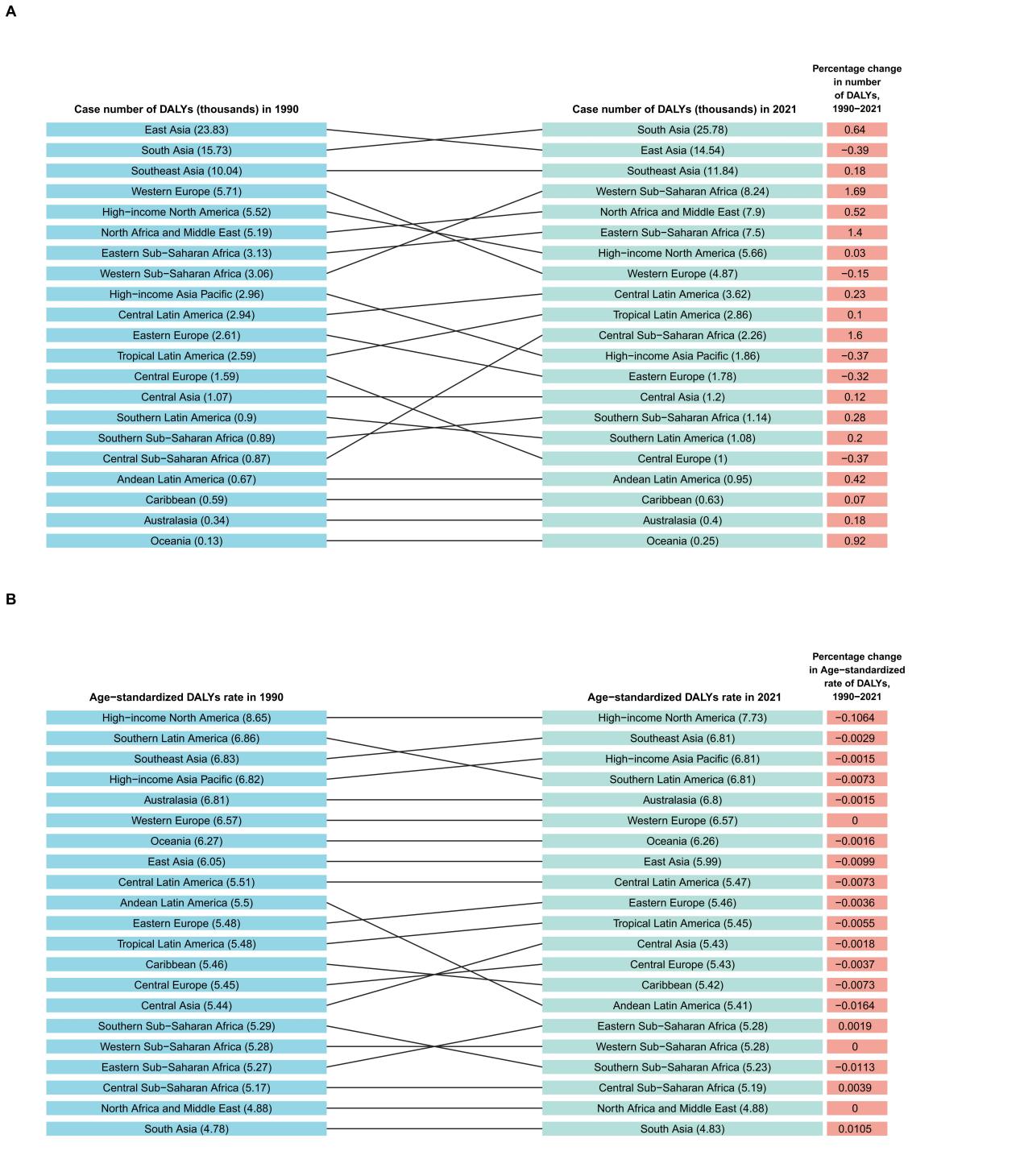


**Figure S5. The number of DALYs (A) and ASDR (B) for alopecia areata among adolescents and young adults across 21 GBD regions from 1990 to 2021.** GBD, Global Burden of Disease; ASDR, Age-standardized DALYs rate; DALYs, disability adjusted life-years.


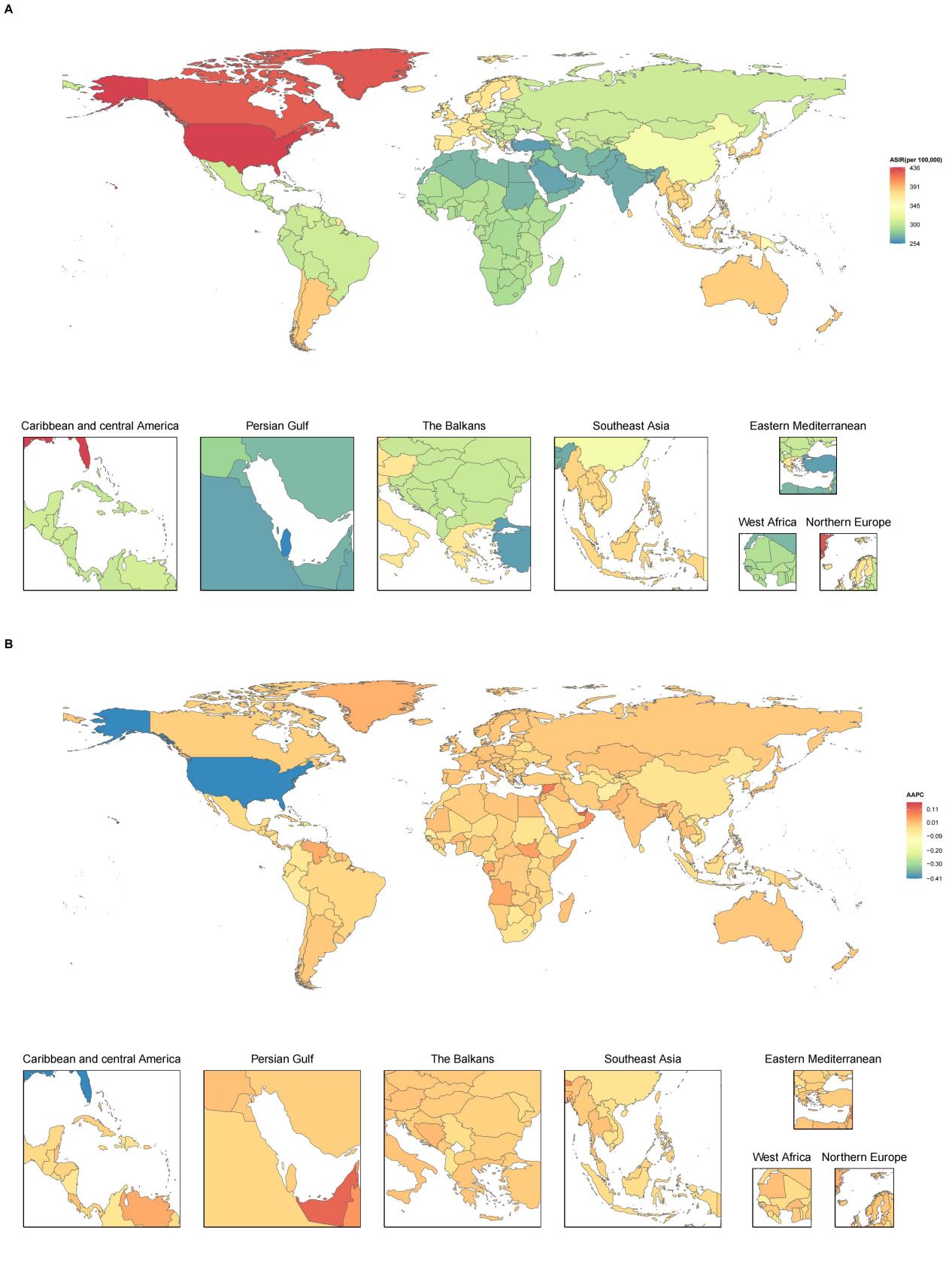


**Figure S6. The global incidence of alopecia areata among adolescents and young adults aged 10-24 years in 204 countries and territories. (A) ASIR in 2021. (B) AAPC in incidence from 1990 to 2021.** AAPC: Average annual percentage change; ASIR, Age-standardized incidence rate.


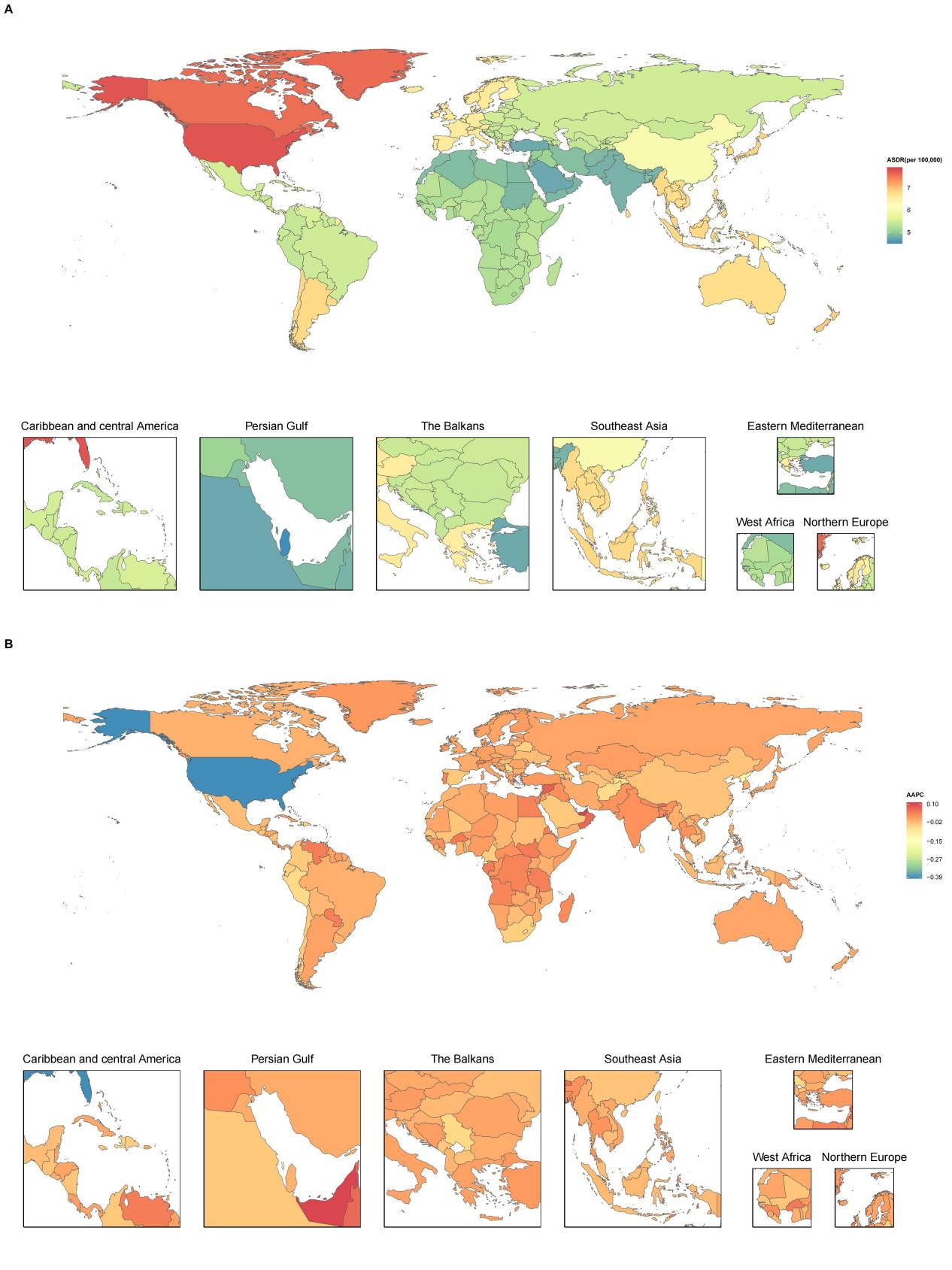


**Figure S7. The global DALYs of alopecia areata among adolescents and young adults aged 10-24 years in 204 countries and territories. (A) ASDR in 2021. (B) AAPC in DALYs from 1990 to 2021.** AAPC: Average annual percentage change; ASDR, Age-standardized DALYs rate; DALYs, disability adjusted life-years.
